# Memory and Proactive Interference for spatially distributed items

**DOI:** 10.3758/s13421-021-01239-1

**Published:** 2022-02-04

**Authors:** Ansgar D. Endress

**Affiliations:** Department of Psychology, City, University of London, Northampton Square, London, EC1V 0HB UK

**Keywords:** Temporary memory, Long-term memory, Working memory, Short-term memory, Proactive interference, Distinctiveness

## Abstract

Our ability to briefly retain information is often limited. Proactive Interference (PI) might contribute to these limitations (e.g., when items in recognition tests are difficult to reject after having appeared recently). In visual Working Memory (WM), spatial information might protect WM against PI, especially if encoding items together with their spatial locations makes item-location combinations less confusable than simple items without a spatial component. Here, I ask (1) if PI is observed for spatially distributed items, (2) if it arises among simple items or among item-location combinations, and (3) if spatial information affects PI at all. I show that, contrary to views that spatial information protects against PI, PI is reliably observed for spatially distributed items except when it is weak. PI mostly reflects items that appear recently or frequently as *memory items*, while occurrences as *test items* play a smaller role, presumably because their temporal context is easier to encode. Through mathematical modeling, I then show that interference occurs among simple items rather than item-location combinations. Finally, to understand the effects of spatial information, I separate the effects of (a) the presence and (b) the predictiveness of spatial information on memory and its susceptibility to PI. Memory is impaired when items are spatially distributed, but, depending on the analysis, unaffected by the predictiveness of spatial information. In contrast, the susceptibility to PI is unaffected by either manipulation. Visual memory is thus impaired by PI for spatially distributed items due to interference from recent memory items (rather than test items or item-location combinations).

## Introduction

Our ability to retain information over brief periods of time is often severely limited, with important consequences in domains ranging from language acquisition (e.g., Baddeley, Gathercole, & Papagno, 1998; Kam & Newport, 2009; Newport, 1990) to educational attainment (e.g., Gathercole, Pickering, Knight, & Stegmann, 2004) to fluid intelligence (e.g., Ackerman, Beier, & Boyle, 2005; Alloway & Alloway, 2010; Engel de Abreu, Conway, & Gathercole, 2010; Fukuda, Vogel, Mayr, & Awh, 2010; Süß, Oberauer, Wittmann, Wilhelm, & Schulze, 2002). However, the reasons for such limitations are debated.

To the extent that what is retained in memory is what is not forgotten, a prominent view is that memory loss is largely determined by interference from other memory items, both in the long-term and in the short-term (e.g., Berman, Jonides, & Lewis, 2009; Keppel & Underwood, 1962; Lewandowsky, Geiger, & Oberauer, 2008; Lewandowsky, Oberauer, & Brown, 2009; Postman & Underwood, 1973; Wickens, Born, & Allen, 1963). In fact, susceptibility to Proactive Interference (that is, impaired memory performance for new information due to pre-existing memory representations) is so closely related to Working Memory (WM) capacity (e.g., Jonides & Nee, 2006; Kane & Engle, 2000; Lustig, May, & Hasher, 2001; May, Hasher, & Kane, 1999; Rosen & Engle, 1998; Rowe, Hasher, & Turcotte, 2010; Shipstead & Engle, 2013) that some authors propose that one of its central functions is to counteract the effects of PI (e.g., Engle, 2002; Nee, Jonides, & Berman, 2007), and others proposed interference-based WM models (e.g., Oberauer, Lewandowsky, Farrell, Jarrold, & Greaves, 2012; Oberauer & Lin, 2017).

However, different forms of WM seem to differ in their susceptibility to PI. In particular, it has been suggested that WM for visual, spatially distributed items might be less susceptible to PI than WM as measured by other paradigms (e.g., Makovski, 2016). Here, I ask four questions related to this apparent discrepancy. First, does PI affect memory for spatially distributed items? Second, are observers sensitive to the strength of PI in spatially distributed items? Third, is the strength of PI determined by PI between simple item representations or between representations comprising both item and spatial information? Fourth, does spatial information *per se* affect memory performance and the susceptibility to PI?

Before presenting the experiments in more detail, it is useful to discuss what is meant by WM. Traditionally, the distinction between WM and Short-Term Memory (STM) is that, while both types of memory operate over relatively brief retention intervals, WM involves some form of active manipulation of the memory items. Active manipulation might include focal attention (e.g., Cowan, 1995, 2001; Engle, 2002; McElree, 2001; Shipstead & Engle, 2013) or removal of distractors (e.g., Ecker, Lewandowsky, & Oberauer, 2014; Oberauer & Lewandowsky, 2016; see also Rosen & Engle, 1998), and might lead to binding among relevant stimulus attributes (e.g., Bateman & Birney, 2019; Oberauer, Süß, Wilhelm, & Wittmann, 2008). However, these short-lived forms of memory might or might not be separable from Long-Term Memory (e.g., Brown, Neath, & Chater, 2007; Ranganath & Blumenfeld, 2005). Further, one of the hallmarks of active manipulation — interactions with attention — is not specific to WM can be observed both with LTM and in infancy (e.g., Fan & Turk-Browne, 2016; Mitsven, Cantrell, Luck, & Oakes, 2018), and the distinction between WM and STM is not always made in the visual WM literature to begin with. Here, I thus use the more neutral label “Temporary Memory” (Endress & Potter, 2014a) because some of the paradigms discussed below are described as visual “WM” paradigms. However, I am not aware of direct evidence that items in such paradigms are actively manipulated, and the specific paradigm studied here most likely relies on the initial and short-lived phases of LTM (Endress & Potter, 2014b). I thus refer to Temporary Memory as a form of memory with a limited life span, but that might or might not involve active manipulation of items and might or might not be separable from LTM.

### Memory limitations and proactive interference

Broadly speaking, PI describes the impairment of memory by prior experience. It has been observed in a variety of paradigms, and can thus have a variety of sources. For example, Oberauer, Awh, and Sutterer (2017) distinguish five main paradigms revealing PI, and in which PI emerges either from confusion among items within WM, or from reduced ability to use Long-Term Memory (LTM) to solve WM tasks.

The oldest paradigm is probably the build-up and release from PI paradigm (e.g., Gardiner, Craik, & Birtwistle, 1972; Kincaid & Wickens, 1970; Wickens et al.,, 1963; Wickens, 1970; Hopkins, Edwards, & Cook, 1972). In this paradigm, participants receive lists of memory items from the same category (e.g., consonants or animals) and have to recall them. The classic finding is that performance decreases as more blocks from the same category are added, but improves when the category is changed. According to Oberauer et al., (2017), in such experiments, WM partially draws on LTM, but that prior experience with the items from the same category diminishes the usefulness of LTM.

Another paradigm is the two-list paradigm (e.g., Tehan & Humphreys, 1996). On each trial, participants learned two lists and then completed a free recall test or probed recall test for the *second* list. On critical trials, both lists contained an item from a common category (e.g., a dog in List 1 and a cat in List 2), while the lists contained unrelated items on control trials. While inclusion of a common category did not produce PI in the free recall task, it did create interference in the cued recall test. For example, when instructed to recall an animal, participants might recall the animal from the incorrect list. Such results suggest that PI can occur during retrieval, either due to confusion in LTM (or within WM if WM is not cleared after the first list has been presented).

The third class of paradigms involves manipulations of temporal distinctiveness (e.g., Brown et al.,, 2007; Shipstead and Engle, 2013). If the temporal context of memory items is less distinct, memory performance is impaired. This impairment might reflect the contribution of episodic LTM to WM tasks: If temporal binding is poorer in episodic LTM, it is less useful for WM tasks as well, even though temporal bindings might also be confusable within WM if the items are presented sufficiently quickly. Relatedly, other models (e.g., Dennis & Humphreys, 2001) use timing as well as other information as “context”, and interference might arise if the contexts of items overlap.^1^

A fourth way in which prior experience may interfere with subsequent learning is if people learn errors they made on previous trials (e.g., Lafond, Tremblay, & Parmentier, 2010). Relatedly, if memory items are represented as sets of features and share some features, shared features might become misbound to the wrong items (e.g., Oberauer, 2009).

Finally, in the recent probe paradigm, participants view a sample array of items and have to make a decision as to whether items in a test array were part of the sample array. Performance is usually impaired when nearby trials share items, irrespective of whether the items are letters (e.g., D’Esposito, Postle, Jonides, & Smith, 1999; Jonides & Nee, 2006), words (e.g., Craig, Berman, Jonides, & Lustig, 2013) or line drawings of animals (e.g., Loosli, Rahm, Unterrainer, Weiller, & Kaller, 2014). In such cases, PI might arise at different stages. First, it might arise at encoding if people are less likely to encode the source of an item if it has a previous source (similar to how prior associations can block subsequent learning; see e.g., Kamin, 1969; Wagner, Logan, Haberlandt, & Price, 1968). Second, it might arise at retrieval if LTM traces compete with WM traces. Third if LTM and WM are dissociable, PI might arise at a decision stage, when participants need to decide between a familiarity signal coming from LTM and a signal coming from WM.

In sum, while PI might occur within WM (e.g., due to confusion among WM items), most of Oberauer et al.,’s (2017) accounts attribute PI to the interaction between LTM and WM, either because PI reduces the usefulness of LTM to WM tasks, because prior learning makes useful associations less likely to be encoded, or due to a competition between LTM representations and WM representations, either at the encoding or the retrieval state.

The view that PI might arise from the interaction between WM and LTM is particularly plausible because it has long been recognized that, in addition to intra-experimental information, information learned previously in our life might create PI as well (e.g., Underwood & Postman, 1960). For example, we might need to type in a phone number, but have a lifetime of experience with number sequences (e.g., other phone numbers, credit card numbers, lottery numbers, …) that might plausibly interfere with our ability to remember yet another number sequence. However, to the extent that WM is truly separable from LTM (see e.g. Brown et al.,, 2007; Ranganath & Blumenfeld, 2005), similar effects can be clearly be observed in WM as well, including release from PI effects (Carroll et al., 2010; Hanley & Scheirer, 1975) and temporal distinctiveness effects (Shipstead & Engle, 2013).

Here, I am agnostic as to how exactly PI arises. Given that the mere presence of interference from other items in memory mathematically guarantees limited memory capacities under fairly general conditions (Endress & Szabó, 2017), I will only conclude that some associations enable searches for memory items, irrespective of whether they are stored in LTM or WM. That is, upon seeing a test item, participants need to perform some kind of memory retrieval to determine whether the item occurred in the current or previous trials, but I am agnostic as to whether they use temporal recency signals, a context signal in terms of other items or any other strategy. In principle, PI might also arise during maintenance, though it is unclear if participants can engage active maintenance mechanisms in fast-paced experiments such as the ones reported below.^2^

In a recent demonstration of the importance of the effects of Proactive Interference (PI) on our ability to briefly retain information, Endress and Potter (2014a) presented participants with rapid sequences of pictures of everyday objects; following each sequence, they viewed another picture and had to decide if it had been part of the sequence. When the pictures were trial-unique and never repeated across trials (in the *Unique Condition*), Endress and Potter (2014a) observed no memory capacity limitations: the probability of encoding any single one of the sequence items was relatively independent of the number of items in a sequence, at least for larger set-sizes. In a marked contrast, when items were drawn from a limited pool of items and reused across trials (in the *Repeated Condition*), performance was much lower and memory capacity estimates remained in the range previously reported.^3^ Repeating items across trials creates PI; for example, it is relatively hard to reject a test item we have seen on recent trials, compared to rejecting a test item that has never occurred at all. These results thus suggest that PI can massively impair memory performance.

However, some visual WM paradigms seem remarkably insensitive to PI. For example, in the change detection paradigm (Luck & Vogel, 1997; see also see also Bays, Catalao, & Husain, 2009; Bays & Husain, 2008; Fukuda et al.,, 2010; Hartshorne, 2008; Jiang, Olson, & Chun, 2000; Lin & Luck, 2012; Makovski, 2016; Makovski & Jiang, 2008; Pertzov & Husain, 2014; Rouder et al.,, 2008; Schneegans & Bays, 2016; Treisman & Zhang, 2006; Zhang & Luck, 2008, among many others), observers view an array of objects (e.g., colored squares); after a delay, they view another array of objects (or a single object in some versions of the paradigm) and have to report whether or not the test array changed with respect to the sample array.

Critically, at least in principle, memory performance in this paradigm might suffer greatly from PI, as an extremely limited set of items is reused over many trials; for example, in Luck and Vogel’s (1997) original experiment, just 7 colors were used over hundreds of trials. Surprisingly, however, the effects of PI seem to be fairly weak in change detection experiments (e.g., Hartshorne, 2008; Lin & Luck, 2012; Makovski & Jiang, 2008). For example, a measure of PI consists in comparing performance in trials with strong PI from immediately preceding trials and in trials with weaker background PI, where PI is still substantial because the same pool of items was reused in all trials, but less strong than when items had occurred in immediately preceding trials (e.g., Hartshorne, 2008; Lin & Luck, 2012; Makovski & Jiang, 2008). Results showed little additional PI when items had occurred in immediately preceding trials.^4^ Even in a large-scale study with thousands of participants, Balaban, Fukuda, and Luria (2019) observed little change in performance across trials in a color change detection task, suggesting either that this paradigm seems relatively insensitive to PI across trials or that, with sufficiently strong PI and highly familiar materials, PI effects arise so quickly that no change in performance is detectable over trials (see e.g., Carroll et al.,, 2010; Gardiner et al.,, 1972; Hopkins et al.,, 1972; Jitsumori, Wright, & Shyan, 1989; Kincaid and Wickens, 1970; Wickens et al.,, 1963; Wickens, 1970, for evidence that PI effects arise very quickly).

### Why are change detection WM experiments so insensitive to PI?

What might explain such differences? I will now discuss five possibilities: Visual WM might be less sensitive to PI than verbal or conceptual WM, some visual WM paradigms might simply be less sensitive to PI than others, PI effects might be limited by background PI, some visual WM paradigms might elicit specific response strategies, or “memory performance” in some visual WM paradigms might not really reflect a memory mechanism but rather the limitations of attentional encoding.

#### Is visual WM less sensitive to PI than other forms of WM?

One possibility is that visual WM is independent from other forms of memory and has different properties. In fact, it is widely accepted that visual WM is at least partially dissociable from verbal WM (e.g., Baddeley, 1996; Cortis Mack, Dent, & Ward, 2018; Cowan, Saults, & Blume, 2014; Oberauer, Süß, Schulze, Wilhelm, & Wittmann, 2000). If change detection tasks fall on the visual side of this spectrum while Endress and Potter’s (2014a) task as well as the recent probes task (D’Esposito et al., 1999; Jonides & Nee, 2006) fall on the verbal side, it is at least possible that verbal WM might be more susceptible to PI than visual WM.

This possibility is particularly plausible because of the stimuli used in these experiments. For example, most of Endress and Potter’s (2014a) experiments used visual material in the form of meaningful pictures. Such pictures are likely encoded in terms of their conceptual meaning rather than its visual properties (e.g., Potter, 1976; Potter, Staub, & O’Connor, 2004; Potter, 2010), which is independent of verbal processes in turn (Endress & Potter, 2012), and meaningful stimuli are easier to remember than other stimuli both in verbal and visual WM (e.g., Brady, Störmer, & Alvarez, 2016; Hulme, Maughan, & Brown, 1991; but see Quirk, Adam, & Vogel, 2020). They also have more stable representations (e.g., Shulman, 1970; Potter et al.,, 2004), to the extent that we have only an extremely limited memory for items that cannot be categorized (e.g., Feigenson & Halberda, 2008; Olsson & Poom, 2005; see also Lewandowsky, 2011; Lewandowsky, Yang, Newell, & Kalish, 2012, for evidence that categorization ability is related to WM). However, memory items can only interfere with other memory items insofar as they are present in memory; as a result, memory items with stronger memory representations might be more likely to interfere with other memory items (though one might also predict reduced interference, for example if stronger memory representations also have more precise encodings of their temporal context).

Be that as it might, if this possibility is correct, change detection experiments likely target fairly low-level visual representations that might not participate in the general cognitive processes that are presumed to be subserved by WM (e.g., Engle, 2002; Conway, Kane, & Engle, 2003; Cowan, 2005; Fukuda et al.,, 2010; Süß et al.,, 2002). In this case, however, it would be difficult to explain why visual WM does predict general cognitive performance (e.g., Fukuda et al.,, 2010).

#### Are change detection experiments less sensitive to PI than other WM paradigms?

A second and related possibility is that different WM experiments make different demands on different aspects of WM, and that different aspects of WM differ in how susceptible they are to PI. For example, Oberauer et al., (2000) evaluated no less than 23 WM tasks that differed in the extent to which they relied on storage of information, transformation of information, supervision, coordination as well as in the modality in which the memory items were presented. It is thus possible that different aspects of WM might be differently susceptible to PI. However, while this possibility is plausible, it is unlikely to account for the relative insensitivity of change detection experiments to PI, given that the superficially similar recent probes task (e.g., D’Esposito et al.,, 1999; Jonides & Nee, 2006) tends to show reliable PI effects.

#### Does background PI mask experiment-internal PI?

A third possibility is that the presence of high background PI limits the observable effects of *additional* PI. For example, Shoval, Luria, and Makovski (2019) showed that PI effects are reduced when items come from a single category (such as faces or houses) compared to when they come from multiple categories. Given that the use of items from the same general category is well known to create PI within just a few trials (see, among many others, Carroll et al.,, 2010; Gardiner et al.,, 1972; Hopkins et al.,, 1972; Jitsumori et al.,, 1989; Kincaid and Wickens, 1970; Wickens et al.,, 1963; Wickens, 1970), Shoval et al.’s (2019) single category conditions compared a high PI condition to a condition with even higher PI. Given that items in change detection experiments typically come from an extremely limited set of items, the presence of strong background PI might thus reduce the observable effects of additional PI.

#### Does change detection performance reflect attentional encoding?

A fourth possibility is that the processes underlying change detection performance really reflect attentional encoding rather than memory per se (e.g., Tsubomi, Fukuda, Watanabe, & Vogel, 2013; see also Fukuda & Vogel, 2009; Fukuda & Vogel, 2011). For example, “WM” limitations can be observed even when objects are in full view. Tsubomi et al., (2013) found similar change detection performance irrespective of whether the sample array was followed by a blank retention interval or whether the sample array remained visible until the test item. Likewise, change detection performance correlates with the maximum number of items people can apprehend without counting (i.e., their subitizing range; Piazza, Fumarola, Chinello, & Melcher, 2011), which, in turn, are thought to be related to attentional processes (e.g., Trick & Pylyshyn, 1994).

Such results are consistent with the classic view that WM items are really those memory items we can pay attention to (e.g., Cowan, 2005). A theory based on attentional encoding limitations also predicts that such limitations predominantly arise for simultaneously presented items (as in change detection tasks) rather than sequentially presented items (as in Endress and Potter 2014a) task). However, unless this form of attentional encoding is disconnected from actual memory processes, such a theory does not necessarily explain why simultaneously presented items are less susceptible to PI than sequentially presented items.

#### Do spatially distributed items elicit specific processing strategies?

A final possibility is that change detection experiments elicit specific processing strategies because memory items are spatially distributed. Such strategies might take three forms: First, participants might use the spatial locations of the items to make them more distinctive across trials (Makovski, 2016); in other words, the relevant memory representations might encode item-location combinations rather than simple items. Second, participants might encode entire spatially organized arrays of items as objects to be stored in memory, or encode their global organization (e.g., Brady & Alvarez, 2015). Third, when facing a difficult memory retrieval problem, participants might attempt to retrieve full encoding episodes from (episodic) long-term memory which contain spatial information in turn. This idea is similar to the presumed role of semantic information in verbal short-term memory. Once (phonological) memory traces fade away, participants might rely on more stable representations from (semantic) long-term memory to solve the retrieval problem (e.g., Hulme et al.,, 1991; Saint-Aubin & Poirier, 1999). In the case of change detection experiments, these more stable representations might be full encoding episodes that comprise spatial information in addition to item representations.

I will now discuss how spatial information might assist in short-term memory retrieval, and return to the other possibilities in the discussion.

### PI and spatial organization

One possible reason for the relative insensitivity of change detection experiments to PI is that such experiments typically use spatially organized stimuli, while other WM experiments tend to use sequentially organized stimuli. For example, Makovski (2016) attributed the relative insensitivity to PI of change detection experiments to automatic bindings between objects and their spatial locations (e.g., Jiang et al.,, 2000; Makovski & Jang, 2008; Pertzov & Husain, 2014; Treisman & Zhang, 2006; Udale, Farrell, & Kent, 2017). For instance, if a dog at Location 1 is encoded separately from the same dog at Location 2, the item-location combinations are (more) trial-unique and thus reduce PI across trials.

Such a strategy is rather plausible. In fact, at least in the case of long-term memory, (mentally) placing memory items in spatial locations such as rooms in a house is a common memory strategy in classical rhetoric (Yates, 1966; though temporal contexts are effective as well; Bouffard, Stokes, Kramer, & Ekstrom, 2018).

Further, in some experiments, spatial information seems to be tightly linked to featural information. Participants confuse features of items that share spatial locations. For example, if they have to remember the orientation of a colored bar, the orientations of bars sharing their spatial positions tend to get confused; in contrast, no such confusion arises when items share non-spatial features such as color (Pertzov and Husain, 2014).

That being said, observers predominantly encode the *relative* spatial positions of objects (e.g., Jiang et al.,, 2000; Treisman & Zhang, 2006; Udale et al.,, 2017). In fact, in change detection experiments using colored shapes as stimuli, an effect of spatial congruency between the sample and the test array emerges only when *all* objects are presented during test, but not when a single test object is shown (e.g., Treisman & Zhang, 2006; Udale et al.,, 2017). Further, spatial information seems to be linked to entire objects rather than object features.^5^ While spatial information is clearly encoded in memory, its usefulness thus seems to depend on the specific paradigm.

To test whether the relative insensitivity of change detection tasks to PI might be due to their spatial organization, Makovski (2016) first replicated Endress and Potter’s (2014a) experiments where all memory items were presented at the center of the screen and observed sizable PI effects. In contrast, spatial cues reduced the strength of the PI effect. Specifically, when, in his Experiment 3, all sample items were presented simultaneously on an imaginary circle (instead of being presented sequentially at the center of the screen), he found the PI effect only for Set-Size 8, but not for Set-Size 4. Likewise, when, in his Experiment 4, items were presented sequentially but with unique spatial positions on an imaginary circle, the PI effect was completely abolished.

In contrast, and in line with the interpretation that binding memory items to spatial positions makes the position-item combinations more distinct and thus reduces PI, a PI effect reemerged if items were presented sequentially on a circle, but if the test item was presented at the center of the screen (Footnote 2 in Makovski, 2016), presumably because this manipulation abolished the usefulness of the spatial cues.

Makovski’s (2016) results thus suggest that spatial cues can reduce PI among memory items even when participants do not *need* to encode spatial information to solve the memory task (though he generally observed PI effects even when items were spatially distributed, at least for larger set-sizes in Experiments 3 and 5). Possibly, spatial cues create more distinct memory representations if participants automatically encode item-location combinations rather than representations of simple items. However, these results are unlikely to explain the marked difference in sensitivity to PI between change detection experiments and the vast majority of other WM paradigms. In fact, Makovski (2016) found considerable PI in what is arguably the most prototypical form of change detection experiments, namely when items were presented *simultaneously* at different spatial locations. In contrast, with the same set-size and a *smaller* total number of items, Balaban et al., (2019) found virtually no evidence for PI in a color change detection experiment (but see the alternative interpretation above).

Further, there were other differences between Makovski’s (2016) and Endress and Potter’s (2014a) experiments that might have decreased the sensitivity to PI in the former experiments. While Makovski (2016) used relatively small set-sizes of up to 8 items drawn from a total pool of 21 items, Endress and Potter (2014a) used set-sizes of up to 20 items in those experiments where they used a total pool of 21 items. Items were thus repeated much more frequently in Endress and Potter’s (2014a) experiments. In the next section, I show that these differences lead to a situation that is equivalent to low-PI conditions in other WM experiments such as the recent-probes task (e.g., Craig et al.,, 2013; D’Esposito et al.,, 1999; Jonides & Nee, 2006; Loosli et al.,, 2014).

### PI and waiting time

While Makovski’s (2016) results might suggest that spatial cues can reduce PI in WM for real-world objects, these results were confounded by the relative low set-sizes. In fact, reducing the set-size while keeping the total pool-size constant increases the mean waiting time between two occurrences of the same item. As an increasing delay between repeated occurrences of an item reduces PI (e.g., Loess & Waugh, 1967; Kincaid & Wickens, 1970; Peterson & Gentile, 1965; Shipstead & Engle, 2013), this effect might have contributed to the relative insensitivity to PI of Makovski’s (2016) experiments when items were spatially distributed.^6^

Specifically, for a set-size of *S* pictures presented on each trial and a total pool-size of *T* pictures presented in the entire (block of an) experiment, the probability that a given picture appears as a sample (in a sample sequence or in a sample array) is *p* = *S*/*T*. As shown in Appendix 1, the probability of a lag of *N* trials between successive occurrences of a sample picture is *p*(1 − *p*)^*N*^, the probability of waiting *at least* *N* trials is (1 − *p*)^*N*^, and the mean lag is $\frac {1}{p} - 1$ trials.

As shown in Appendix A, only waiting times between occurrences of an item as a sample depend on the set-size, while the probability of occurrence as a test item does not. Critically, as observers can accumulate long-term memory traces from repeated exposure to even briefly presented items (e.g., Endress & Potter, 2014b; Melcher, 2001; Melcher, 2006; Pertzov et al.,, 2009; Thunell & Thorpe, 2019), it seems plausible that a reduced waiting time between consecutive occurrences of an item might increase PI.

**Fig. 1:**
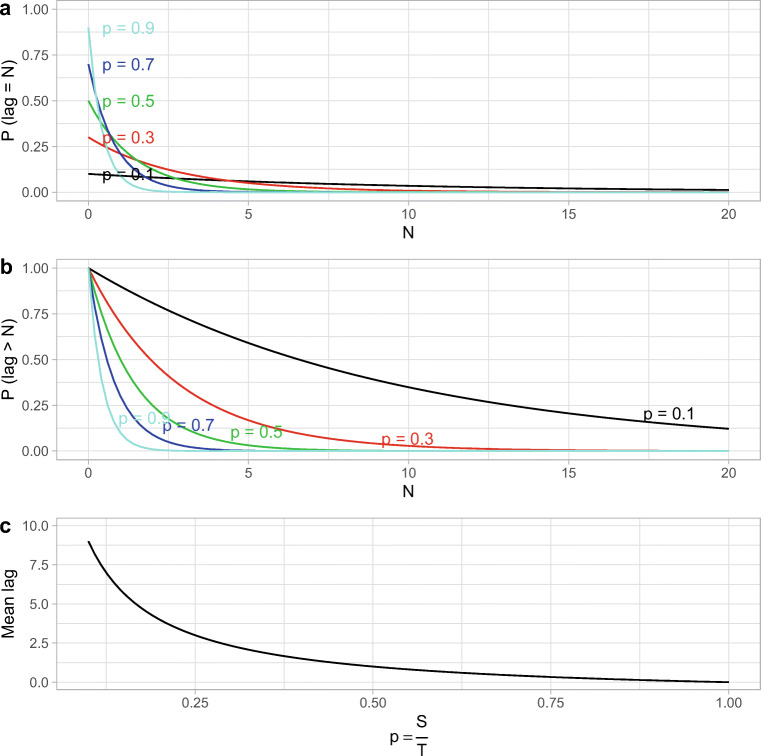
(a) Probability of a lag of *N* trials between two successive occurrence of an item as a sample. The different curves show different ratios between the set-size and the size of the total pool of items. The combinations of set-sizes and pool-sizes in Makovski’s (2016) experiments correspond to *p* = 4/21 ≈ .19 and *p* = 8/21 ≈ .38, respectively. (b) Probability of waiting for *at least* *N* trials between two occurrences of an item as a sample item. Again, the different curves represent different ratios between the set-size and the total pool-size. (c) Mean waiting time (in trials) between two consecutive occurrences of an item as a sample item as a function of the ratio between the set-size and the total pool-size

As shown in Fig. 1a, the probability of lags greater than, say, 5 trials is small irrespective of the set-size. However, Fig. 1b shows that small set-sizes allow for substantial probabilities of lags of *at least* 5. For example, and as mentioned above, in his Experiments 3 and 4, Makovski (2016) used the set-sizes 4 and 8, with a total Pool-Size for 21 items. This corresponds to probabilities of lags of at least 5 trials of 35% and 9%, respectively (and 19% assuming an average set-size of 6). Figure 1c shows the mean lag between two occurrences of the same item as a sample item. The average lag with set-sizes 4, 6 and 8 is 4.3, 2.5 and 1.6 trials, respectively. At least for the smaller set-sizes, such waiting times are considered low-PI conditions in the recent probes task (e.g., Craig et al.,, 2013; D’Esposito et al.,, 1999; Jonides & Nee, 2006; Loosli et al.,, 2014). As a result, spatially distributed items might well show PI effects when PI is strengthened by reducing the waiting time between subsequent occurrences of an item.

### Spatial information vs. waiting time

Spatial information clearly affects memory performance. This is particularly clear from a comparison of Makovski’s (2016) Experiments 1 and 4. In both experiments, participants viewed sequences of objects and completed a recognition test after each sequence; the only difference between these experiments was that, in Experiment 1, all items were presented at the center of the screen while in Experiment 4, items were presented on an imaginary circle. Still, PI effects were observed only in Experiment 1 but not in Experiment 4.^7^

However, it is unclear *why* spatial information affects PI. In fact, if spatial information were the main source of information that eliminates PI among items, PI should not only be reduced with sequentially presented items, but also with simultaneously presented items, as long as they are spatially distributed. However, in his Experiment 3, Makovski (2016) found reliable PI effects for spatially distributed items when they were presented simultaneously rather than sequentially (though the waiting time between two occurrences of the same item was much reduced compared to Experiment 4 due to the fast presentation speed).

One possibility suggested by Makovski (2016) is that observers encode item-location combinations (e.g., a dog at 9h). However, it is not clear if encoding item-location combinations would be a viable memory strategy, for two reasons. First, observers predominantly encode *relative* spatial positions of objects (e.g., Jiang et al.,, 2000; Treisman & Zhang, 2006; Udale et al.,, 2017) rather than the absolute ones required for item-location combinations. Second, in everyday cognition, such a strategy would be most useful for long-term retention of inanimate objects rather than for a general WM system used in moment-to-moment reasoning, given that objects tend to change location, especially if they are animate. In line with this view, item-information is more stable over time than location-information in probabilistic foraging tasks (e.g., Téglás et al.,, 2011) and the two types of information have dissociable neural substrates (e.g., Goodale & Milner, 1992; Mishkin and Ungerleider, 1982).

An alternative view is that the presence of spatial information changes the response strategies rather than the underlying representations. For example, participants might use the spatial information provided during test to initiate a memory search. To use an analogy with face recognition, similarly to the *Unique Condition* in the absence of spatial cues, it is relatively easy to discriminate faces of friends from faces of strangers, while, similarly to the *Repeated Condition* in the absence of spatial cues, it is harder to discriminate faces of good friends we have seen very recently from faces of good friends we have seen somewhat less recently.

In contrast, introducing additional retrieval cues changes the task demands. For example, we might try to discriminate faces of individuals who were at some party from individuals who were not. In this case, it is possible to initiate a memory search through our friends to decide who was at the party and who was not, though this search would become much more difficult when most of our friends are party animals who attend most parties. In contrast, discriminating previously unknown partygoers from complete strangers should still be possible, even though it might be harder to link a stranger to a party than a friend due to the lack of familiarity. In other words, changing the task demands by introducing retrieval cues might reduce the gap between the Unique and the *Repeated Condition*, unless interference in the *Repeated Condition* becomes too strong to make memory search feasible.

As mentioned above, the latter idea is similar to the possible role of semantic information in verbal short-term memory. When unstable memory traces fade away, participants might rely on more stable representations, and these come from (semantic) long-term memory (e.g., Hulme et al.,, 1991; Saint-Aubin & Poirier, 1999). In Makovski’s (2016) experiments, the long-term memory traces might be full encoding episodes that comprise spatial information.

These views make different predictions, because they make different assumptions about the total pool of possible items. If participants encode item-location combinations, the effective total pool-size is the number of locations (i.e., the set-size) multiplied with the total number of items, *S* × *T*. As a result, the mean waiting time between two occurrences of the same item-location combination is *T* − 1 and does not depend on the set-size.^8^^,^^9^

In contrast, if the participants use spatial information just as a retrieval cue, the critical total pool is still that of the available items, and the mean waiting time between subsequent occurrences of an item is $\frac {T}{S} - 1$.^10^

If participants encode item-location combinations, they should thus be relatively insensitive to the ratio between the set-size and the total pool-size; in contrast, if the predominant memory representations are item-based, the critical determinant of the strength of PI is the ratio between the set-size and the total pool-size.

Similar predictions follow if PI does not depend on the time since an item has been observed, but on rather the total number of times it has been observed. If the relevant pool is the number of (simple) items, then the expected number of occurrences per trial of any item is given by *S*/*T*. In contrast, if the relevant pool is the number of item-location combinations, the expected number of occurrences per trial is *S*/(*S* × *T*) = 1/*T*. As before, participants should be sensitive to the set-size only if they maintain memory representations of simple items, but not if they keep memory representations of item-location combinations.

## The current experiments

In the experiments below, I ask three questions. First, does PI affect memory for spatially distributed items? Second, is the strength of PI determined by PI between simple item representations or between representations of item-location combinations, and does spatial information *per se* affect memory performance and the susceptibility to PI? Third, does either waiting time or spatial information explain the differences in susceptibility to PI between change detection experiments and other WM paradigms?

In Experiment 1, I ask whether the strength of PI affects memory performance when memory items are spatially distributed. In each trial, participants viewed a sequence of 8 items, presented sequentially on an imaginary circle; below, I will call the number of memory items the *Set-Size*. Following this, they viewed another (test) item and had to indicate whether or not this latter item had been part of the sequence. Critically, in different blocks, I varied the size of the total pool from which items could be drawn: Items came from (1) a pool of 9 items in total, (2) a pool of 22 items in total or (3) were trial-unique (*Pool-Size*= *∞*), respectively. Given that items are repeated more often when there are fewer items, I expected greater PI for smaller pool-sizes.

To preview the results, I found substantial PI for Pool-Size 9, but not for the larger Pool-Size 22, where performance was similar to the *Unique Condition*. Participants are thus sensitive to the strength of PI among spatially distributed items. However, these results are ambiguous as to whether PI occurs among the representations of simple items or of item-location combinations.

This ambiguity is addressed in Experiment 2 (and in a pilot experiment reported in Appendix B). In Experiment 2, I used a constant pool-size similar to the pool-sizes for which neither Makovski (2016) nor Experiment 1 above detected PI effects among spatially distributed items. Critically, however, I increased the set-size. As mentioned above, if people maintain representations of item-location combinations, the strength of PI should be unaffected by the set-size; if they maintain representations of simple items, the strength of PI should be determined by the ratio between the set-size and the total number of (simple) items. Hence, if participants encoded item-location combinations, they should be insensitive to PI as in Experiment 1 and Makovski’s (2016) experiment. In contrast, if location information is extraneous (possibly an extra retrieval cue from episodic long-term memory), participants should be sensitive to PI.

Specifically, participants in Experiment 2 completed a block where pictures were trial-unique and one where they were sampled from a total pool of 21 pictures. Within each block, participants viewed sequences of 12 or 20 images that were presented on an imaginary circle, proceeding in a clockwise direction. The set-size was thus increased compared to Experiment 1. (In the Pilot Experiment, the Set-Size was either 8 or 16, while the Pool-Size was 17.)

To preview the results, I found significant PI under these conditions, suggesting that participants were sensitive to the ratio between the set-size and the total number of items and thus that the strength of PI is determined by the waiting time between subsequent occurrences of simple items rather than item-location combinations.

In Experiment 3, I ask whether spatial information *per se* has an effect on memory performance and susceptibility to PI. Participants viewed sequences of 15 items, followed by a single item test image. In different blocks, the items appeared either (1) at the center of the screen, (2) on an imaginary circle with items proceeding in a clockwise direction or (3) on an imaginary circle where locations were chosen in a random order. Within each block, there was a sub-block where items were *trial-unique* and a sub-block where items were repeatedly drawn from a total pool of 16 items. I asked if performance differed depending on whether items were spatially distributed and depending on whether participants can attentionally anticipate where an item will appear.

To preview the results, I found that spatially distributing items impaired memory but did not affect the strength of PI; in contrast, the predictability of item location affected neither memory nor the strength of PI.

## General methods

### Participants

While I had no specific predictions regarding the required sample size for the current experiments, I targeted 30 participants per experiment (or the closest multiple of the number of counterbalancing conditions; see below); this sample size was choosen as it was slightly larger than the samples in Endress and Potter’s (2014a) experiments, which yielded reliable PI effects. However, in Experiment 3, I decided to double the sample size as the effect sizes were too small to draw clear conclusions from the smaller sample.

Before analyzing the data, I excluded those participants who were unlikely to have paid attention to the task. I note that these criteria are not related to the participants’ performance, but were applied before any analyses were performed. Based on prior experience with the current participant population, I expected to have to exclude roughly a third of the sample due to insufficient attention to the task (e.g., chance performance in trivial control tasks). As the objective of the current experiment is to provide evidence for PI, I did not exclude participants with low performance in the *Unique Condition*. As participants who perform well in the *Unique Condition* have more room to show PI, excluding participants based on their performance in the *Unique Condition* might inflate the estimate of the strength of PI. Given that some participants might show genuinely low performance, it is more conservative *not* to exclude participants based on their performance.

I first excluded participants who took excessively long to complete the experiment. This criterion was based on the observation that some participants were observed (through a window in the closed door of the testing room) playing with their cellphones or other implements during the experiment and/or took excessive breaks, which, in turn, is likely to reduce the strength of PI (Loess and Waugh, 1967; Kincaid & Wickens, 1970; Peterson & Gentile, 1965; Shipstead & Engle, 2013). As I was unlikely to have witnessed all such instances, I thus excluded participants based on the time it took them to complete the experiments. I thus excluded participants who were slower than 3 standard deviations from the mean duration for an experiment, leading to the exclusion of 4 out of 169 participants.

I excluded another 15 participants for the following reasons: Computer problems (*N*= 4), having been tested previously (*N*= 1), not understanding or misunderstanding instructions (*N* = 2), almost falling asleep (*N* = 1), not looking at the screen throughout the experiment (*N* = 1) and playing with a phone or other implements (*N* = 6).

The final demographic information in the sample is given in Table 1.

**Table 1:** Demographics of the final sample after exclusion criteria have been applied

Experiment	*N*	Females	Males	Mean age	Age range
Exp. 1	30	16	14	27.7	18–43
Exp. 2	30	20	10	23.9	18–57
Exp. 3	60	38	22	23.5	17–45
Pilot Exp.	32	21	11	24.4	18–44

### Apparatus

Stimuli were presented on a Dell P2213 22” (55.88 cm) LCD (resolution: 1680 × 1050 pixels at 60 Hz), using the Matlab psychophysics toolbox (Brainard, 1997; Pelli, 1997) on a Mac Mini computer (Apple Inc., Cupertino, CA). Responses were collected from pre-marked “Yes” and “No” keys on the keyboard.

### Materials

Stimuli were color pictures of everyday objects taken from Brady et al., (2008). These were randomly selected for each participant from a set of 2,400. Stimuli were presented on an imaginary circle with a radius of 190 pixels (except in one condition in Experiment 3, as noted below), corresponding to a viewing angle of 5.1 dva at a typical viewing distance of 60 cm.

Images were scaled to the maximal size so that they would not overlap on the circle.^11^ In Experiment 1, items were thus scaled to a size of 130 × 130 pixels (3.5 dva), while items were scaled to a size of 70 × 70 pixels (1.9 dva) in Experiments 2 and 3.

### Procedure

**Fig. 2:**
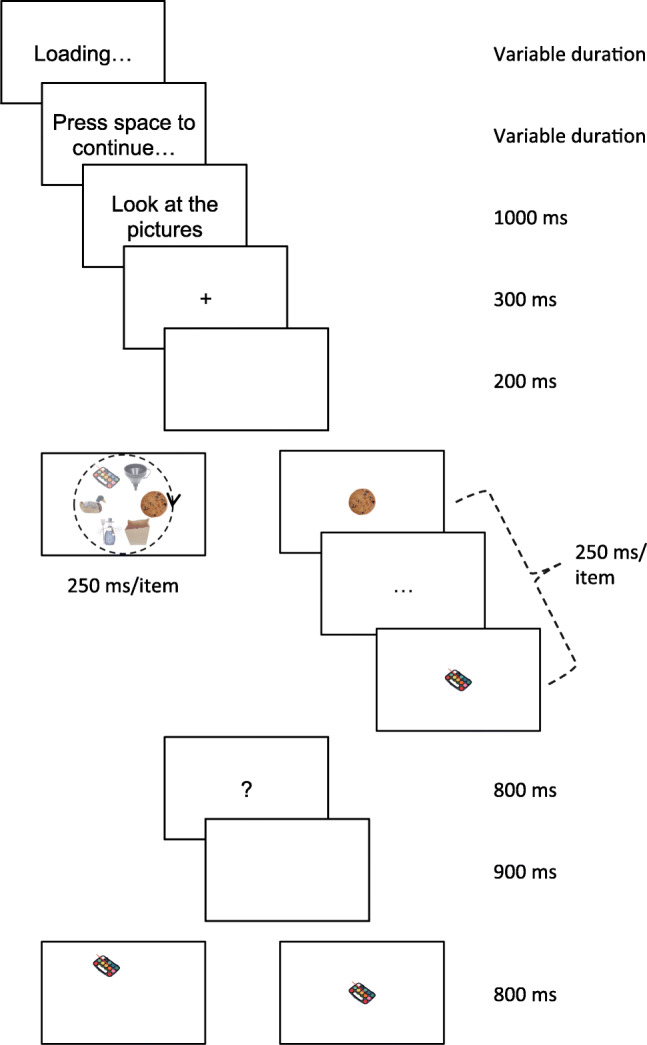
Trial schedule in the different experiments. Trials started with a (usually invisible) screen indicating that the stimuli were being loaded, followed by a button-press to start a trial, a fixation cross, a blank screen and finally the sample sequence. Samples were either presented on an imaginary circle (left) or at the center of the screen (right). After the sample sequence, participants saw a question mark, followed by a blank screen and finally the test item. If the test item had been part of the sample sequence, it appeared in its original position

As shown in Fig. 2, trials started with a screen indicating that the stimuli were being loaded for as long as they were being loaded (i.e., in general the screen was invisible). Following this, participants had to press the space bar to continue (except in Experiment 2, where I added an extra 700 ms to the fixation instead), followed by a fixation cross presented for 300 ms, a blank screen presented for 200 ms and then the sample sequence. Sample images were presented for 250 ms each in immediate succession (with no interstimulus interval), either on an imaginary circle or at the center of the screen (*Center* Condition in Experiment 3). When presented on an imaginary circle, the location of the first sample image was randomly chosen.

Following the sample sequence, a blank screen was shown for 900 ms, followed by the test item. Test items were presented for 800 ms but participants had unlimited time to respond.

Test items had appeared in the sequence on half of the trials; on these trials, they appeared in the spatial position in which they had appeared in the sample sequence. “Old” test items were chosen equally often from two sequence-initial, two sequence-medial and two sequence-final positions, respectively. Foil test items that had *not* appeared in the sample sequence were presented at the location corresponding to the sequential positions from which “old” test items had been sampled; given that the first sample started at a random location, foil images thus essentially appeared at random locations as well.

A new trial started immediately after a participant response.

Verbal suppression was not administered because earlier research has shown that neither memory nor PI is affected by verbal suppression at the current presentation rates (Endress and Siddique, 2016).

The research has been approved by the City, University of London, Psychology Ethics Committee (ETH1819-0400).

### Analysis strategy

The analyses below will be based on two types of measures. First, I will seek to analyze performance in terms of (1) memory per se and (2) susceptibility to PI. Second, I will analyze the data in terms of raw accuracy. Analyses in terms of hit and false alarm rates are reported in Appendix D.

#### Memory and susceptibility to PI

Memory per se will be operationalized as the performance in the *Unique Condition*. This condition should be a relatively pure measure of memory performance, as participants just need to encode, store and retrieve items from memory in the absence of interference (other than the fact to have completed other trials with other stimuli).

Susceptibility to PI will be operationalized as the relative Cost of PI (see Endress & Siddique, 2016), defined as

$$ \text{Cost of PI} = \frac{\text{Unique} - \text{Repeated}}{\text{Unique}} $$

This measure gives the relative performance decrement in the *Repeated Condition* compared to the *Unique Condition*, normalized by the performance in the *Unique Condition*. For example, if a participant has an accuracy of 80% in the *Unique Condition* and 60% in the *Repeated Condition*, the relative performance decrement due to PI is (80% – 60%) / 80% = 25%.

As shown in Table 2, some cells in some experiments do not meet the assumption of normality. As my predictions for the first set of analyses involve pairwise comparisons, I seek to apply the same statistical tests across experiments and thus use pairwise Wilcoxon tests in all experiments instead of using Gaussian-based statistics in those experiments where the assumptions are met. However, Gaussian-based statistics would give similar results.

**Table 2:** Cells across experiments where a violation of normality was detected by a Shapiro-Wilk test when performance was measured in terms of accuracy and the Cost of PI, respectively

Experiment	*Set-Size*	*Location Condition*	*PI Condition*	W	*p*
Accuracy					
Exp. 1	8	NA	Unique	0.88	0.002
Pilot Exp.	16	NA	Unique	0.93	0.036
Cost of PI					
Exp. 3	15	Circle - Random	NA	0.953	0.028
Pilot Exp.	16	NA	NA	0.919	0.017

#### Raw accuracy

The second set of analyses involves performance in terms of accuracy in the different conditions, using generalized linear mixed models (GLMMs) treating the accuracy in individual trials as a binary random variable. The advantage with respect to the first set of analyses is that I can jointly analyze memory and susceptibility to PI; the disadvantage is that the measure of susceptibility to PI (i.e., performance in the *Repeated Condition*) is contaminated by contributions from memory per se. The order of the blocks as well as its interactions with the other predictors were initially included as well. However, except in Experiment 3, these predictors did not contribute to the model likelihood and were thus removed from the models (Baayen, Davidson, & Bates, 2008).

## Experiment 1: The role of PI strength

In Experiment 1, I asked if PI effects are more likely to be observed with stronger PI. I manipulated the strength of PI by manipulating the size of the total pool from which stimuli could be drawn. With smaller pools, items are repeated more frequently, which should lead to stronger PI in turn.

### Materials and methods

In all trials, 8 items were presented on an imaginary circle as described above. Critically, across blocks, the size of the pool from which items could be drawn was set to infinite (i.e., in the *Unique Condition*), 22 or 9.

The order of blocks was counterbalanced across participants. A third of the participants completed the experiment in each of the Pool-Size orders *∞–22–9*, *9–22–∞* and *22–∞–9*. Each block comprised 84 trials. Participants could take a break after each block. Before starting the experiment, participants were given two training trials. (There were no training trials in the other experiments.)

### Results

I first analyze the results in terms of the performance in the *Unique Condition* and in terms of the Cost of PI and then in terms of the raw accuracy in the *Unique Condition* and the *Repeated Condition*, respectively.

#### Analyses of memory vs. susceptibility to PI

**Fig. 3:**
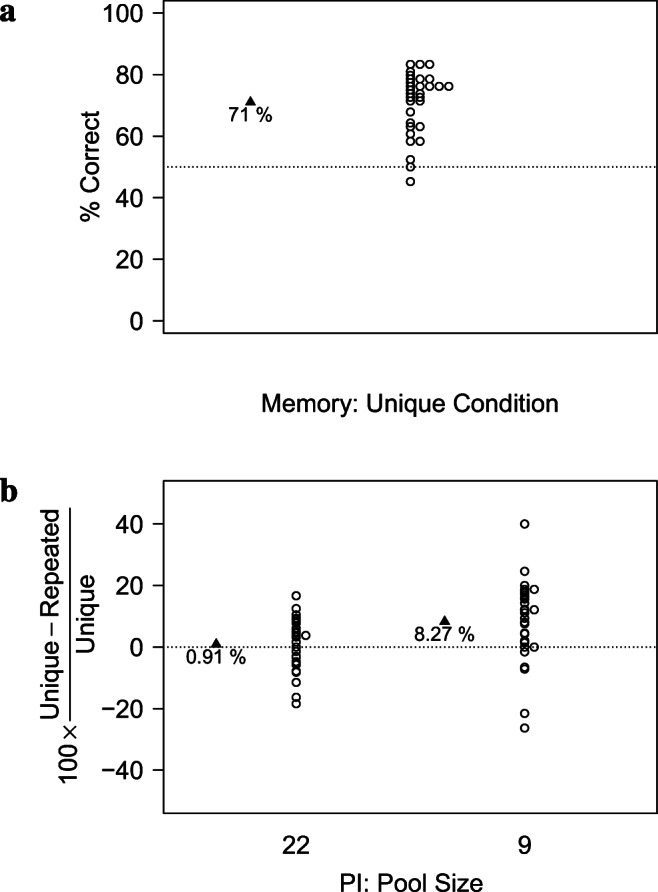
Results of Experiment 1 in terms of the performance in the *Unique Condition* (a) and the relative *Cost of PI* (b)

As shown in Table 3 and Fig. 3, performance in the *Unique Condition* was well above chance.

**Table 3:** Descriptive statistics in terms of raw accuracy and Cost of PI in Experiment 1. *p*_*Wilcoxon*_ indicates the *p* values of a Wilcoxon test against the chance levels of 50% (accuracy) and 0 (Cost of PI), respectively

*Pool-Size*	N	*M*	*SD*	*SE*	*p*_*Wilcoxon*_	Cohen’s *d*
Accuracy						
*∞*	30	0.706	0.102	0.019	<.001	2.03
22	30	0.699	0.099	0.018	<.001	2.02
9	30	0.641	0.087	0.016	<.001	1.62
Cost of PI						
22	30	0.006	0.082	0.015	0.430	0.073
9	30	0.081	0.132	0.024	0.002	0.618

As shown in Table 3 and Fig. 3, the Cost of PI differed from zero only when items came from a total pool of 9 items in total, but not when items came from a pool of 22 items. A paired Wilcoxon Test showed that the Cost of PI differed significantly between *Pool-Sizes* 9 and 22, *V* = 76, *p*= 0.001, *CI*_.95_ = − 0.113, -0.029.

Taken together, these results suggest that PI needs to be sufficiently strong to have a noticeable effect. When, as in Makovski’s (2016) experiments, 8 items were picked from a total pool of 22 items, no PI effects were observed. In contrast, when the total Pool-Size was limited to 9 items, a sizeable PI effect emerged (Cohen’s *d*=.618).

#### Analysis in terms of accuracy

**Fig. 4:**
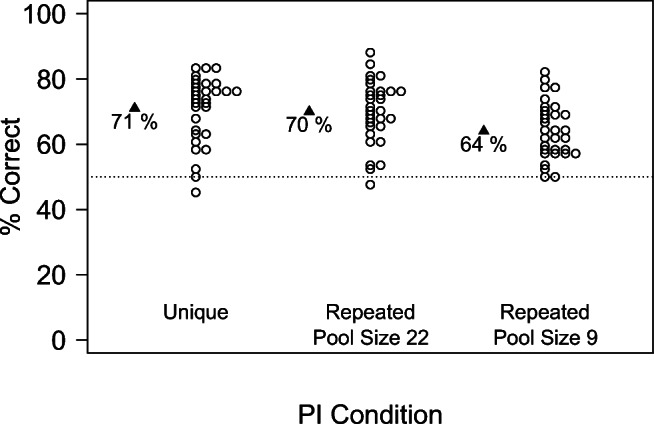
Results of Experiment 1 in terms of raw accuracy. When items were drawn from a total pool of 22 items, performance was undistinguishable from performance in the *Unique Condition*, where items were trial-unique. In contrast, when items were drawn from a total pool of 9 items, a sizeable PI effect emerged

As shown in Fig. 4, performance in terms of raw accuracy was better in the *Unique Condition* than when items were drawn from a total pool of 9 items, while performance was similar in *Unique Condition* and for *Pool-Size* 22.

These results were confirmed in series of Generalized Linear Mixed Models, treating the trial-by-trial accuracy as a binary random variable. I first fit a model with the random factor *Participants* and the fixed factor *Pool-Size*, treating the *Unique Condition* as the reference level. The order of the blocks and its interaction with *Pool-Size* did not contribute to the model likelihood and were thus removed (Baayen et al., 2008). As shown in Table 4, performance was better in the *Unique Condition* than for *Pool-Size* 9, while the *Unique Condition* did not differ statistically from the *Pool-Size* 22 condition.

**Table 4:** Results of a generalized linear mixed model for Experiment 1, with the fixed factor *Pool-Size*, treating the *Unique Condition* as the reference level. Compared to the *Unique Condition*, performance was impaired for Pool-Size 9, but not for Pool-Size 22. The results show two models that include (top) or exclude (bottom) the *Unique Condition*

Effect	Estimate	Std. Error	*CI*	*t*	*p*
Model including the *Unique Condition*					
*Pool-Size* 22	− 0.035	0.062	− 0.158, 0.0873	− 0.563	0.574
*Pool-Size* 9	− 0.307	0.061	− 0.427, − 0.187	− 5.025	< 0.001
Model excluding the *Unique Condition*					
*Pool-Size* 9	− 0.271	0.061	− 0.39, − 0.152	− 4.46	< 0.001

Further, and as shown in Table 4 (bottom), a model fit to the data after removing the *Unique Condition* showed that performance was significantly worse in the *Pool-Size* 9 condition than in the *Pool-Size* 22 condition.

Appendix D.1 shows the results in terms of the hit and false alarm rates. The hit rate was higher in the *Unique Condition* than in the two other *Pool-Size* conditions, while it did not differ between the latter two conditions. The false alarm rate was higher for *Pool-Size* 9 than for *Pool-Size* 22, which had a higher false alarm rate than the *Unique Condition*.

### Discussion

The results of Experiment 1 show that PI effects are readily observed with spatially distributed items when PI is strong enough: When, on each trial, 8 items are shown on an imaginary circle, performance is impaired when items are taken from a limited pool of 9 items compared to when they are trial-unique. In contrast, when items come from a pool of 22 items in total, performance is equivalent to the *Unique Condition*.

Observers are thus sensitive to the strength of PI. However, the results of Experiment 1 are ambiguous as to whether PI occurs among the representations of simple items or rather among representations of item-location combinations. As discussed in the Introduction, these possibilities make different predictions with respect to the role of the set-size. If participants represent simple items, the strength of PI should be determined by the ratio between the set-size on each trial and the total number of possible items. After all, this ratio determines for how many trials we need to wait before seeing a given item again.

In contrast, if people represent item-location combinations, the strength of PI should be determined only by the total number of items but not by the set-size. These possibilities are addressed in Experiment 2 by keeping the total pool-size constant and by increasing the set-size. If participants represent item-location combinations, they should still be insensitive to PI when the total pool-size is kept constant; this is because the waiting time between two occurrences of an item depends only on the total pool-size, but not the set-size. In contrast, if they represent simple items with no spatial component, increasing the set-size should increase the strength of PI, because the waiting time between two occurrences of an item depends on both the set-size and the pool-size.

## Experiment 2: The role of the set-size

In Experiment 2, I asked if PI effects depend on the set-size when items were spatially distributed and when the pool-size is kept constant. As mentioned above, if people use spatial information to resist PI by representing item-location combinations, they should not be sensitive to manipulations of the set-size; as a result, as in Experiment 1 and Makovski (2016), they should not be sensitive to PI with a pool size of 21. In contrast, if people predominantly represent simple items, they should show more PI as the ratio between the set-size and the total pool-size increases.

### Materials and methods

In all trials, participants viewed a sequence of objects, presented on an imaginary circle moving in a clock-wise direction. Participants completed a *Unique Condition* and a *Repeated Condition* (where items were drawn from a total pool of 21 items); the order of these conditions was counterbalanced across participants. On each trial, participants were presented either with 12 or 20 items. The same Set-Size could not occur more than three times in a row. There were 120 trials per PI condition. Participants could take a break every 60 trials.

### Results

I first analyze the results in terms of the performance in the *Unique Condition* and in terms of the Cost of PI and then in terms of the raw accuracy in the *Unique Condition* and the *Repeated Condition*, respectively.

#### Analyses of memory vs. susceptibility to PI

**Fig. 5:**
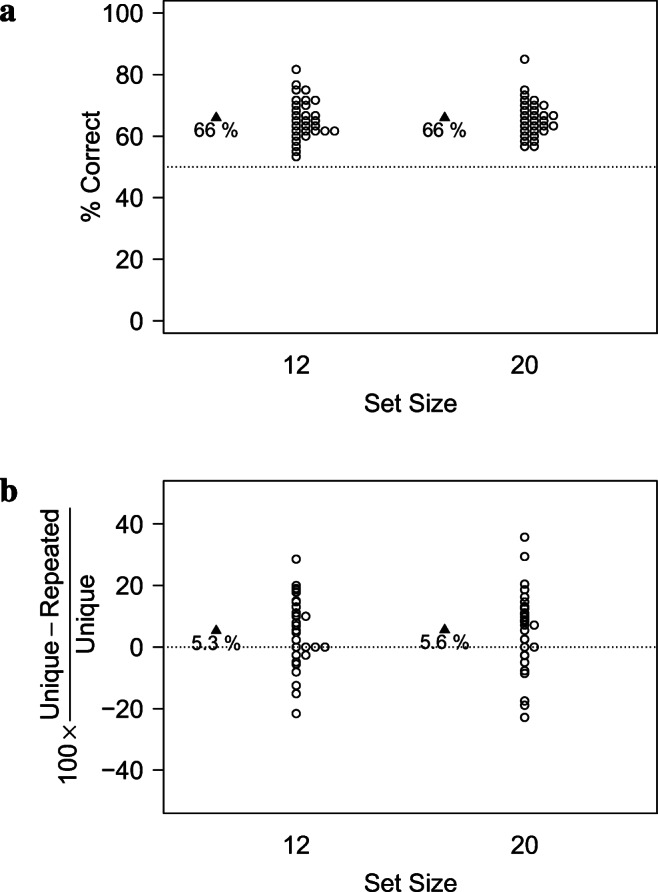
Results of Experiment 2 in terms of the accuracy in the *Unique Condition* (a) and of the relative *Cost of PI* (b). Neither measure appears to be affected by the Set-Size

As shown in Fig. 5 and Table 5, both the performance in the *Unique Condition* and the Cost of PI were well above chance, but neither measure was affected by the set-size. Accordingly, a paired Wilcoxon test revealed no difference between the set-sizes, neither for accuracy in the *Unique Condition*, *V* = 169, *p*= 0.871, *CI*_.95_ = − 0.04, 0.03, nor for the Cost of PI, *V* = 239, *p* = 0.903, *CI*_.95_ = -0.06, 0.06.

**Table 5:** Descriptive statistics in terms of raw accuracy and Cost of PI in Experiment 2. *p*_*Wilcoxon*_ indicates the *p* values of a Wilcoxon test against the chance levels of 50% (accuracy) and 0 (Cost of PI), respectively

*PI Condition*	*Set-Size*	N	*M*	*SD*	*SE*	*p*_*Wilcoxon*_	Cohen’s *d*
Accuracy							
Unique	12	30	0.656	0.066	0.012	<.001	2.36
Unique	20	30	0.658	0.061	0.011	<.001	2.60
Repeated	12	30	0.616	0.057	0.010	<.001	2.05
Repeated	20	30	0.616	0.068	0.012	<.001	1.70
Cost of PI							
–	12	30	0.053	0.116	0.021	0.024	0.461
–	20	30	0.056	0.131	0.024	0.026	0.430

#### Analysis in terms of accuracy

**Fig. 6:**
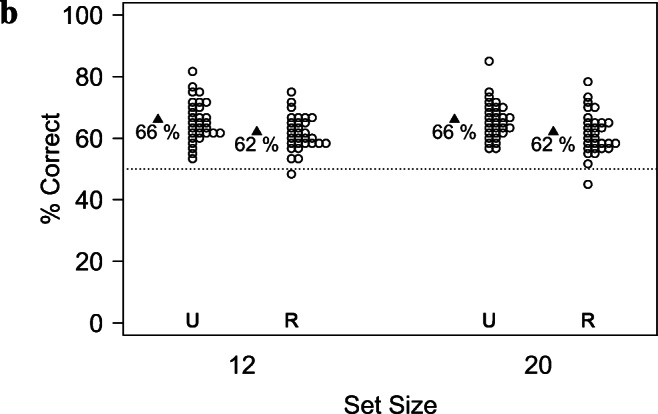
Results of Experiment 2 in terms of raw accuracy, grouped by the *Set-Sizes*. U and R represent the Unique and the Repeated Condition, respectively. A While PI impairs memory performance, memory performance is largely unaffected by the Set-Size

As shown in Fig. 6 and Table 5, performance in terms of raw accuracy differed across the *PI Conditions*, but was unaffected by the *Set-Size*. This was confirmed by a generalized linear mixed model with the within-subject predictors *PI Condition* and *Set-Size*, treating the trial-by-trial accuracy as binary random variables. Following Baayen et al., (2008), I then removed the interaction term from the model as it did not contribute to the model likelihood. I also included the block order as well as its interactions with the other factors, but they did not contribute to the model likelihood. The results are shown in Table 6.

**Table 6:** Results of a generalized linear mixed model for Experiment 2. I treated the *Unique Condition* and a *Set-Size* of 12 as the reference levels for the two predictors

Effect	Estimate	*SE*	*CI*	*t*	*p*
*PI Condition*: Repeated	− 0.177	0.049	− 0.273, − 0.0808	− 3.604	0.000
*Set-Size*: 20	0.004	0.049	− 0.0926, 0.0999	0.074	0.941

While performance was significantly worse for the *Repeated Condition*, the difference between the Set-Sizes was not significant.

To provide evidence for the absence of a set-size effect, I calculated the likelihood ratio in favor of the null hypothesis in the following way. First, for each participant and set-size, I averaged the accuracy, and, for each participant, subtracted the averages for the Set-Sizes from each other. A Shapiro-Wilk test did not detect any deviations from normality for these differences. Then, following Glover and Dixon (2004), I calculated the likelihood ratio of the hypotheses that (1) the differences were not different from zero and (2) that they were. (This approach is similar to an analysis using Bayes factors, except that it is frequentist and does not make arbitrary assumptions about the prior distribution of the effect sizes.)

The null hypothesis was 3.1 times more likely than the alternative hypothesis after correction with the Akaike information criterion, and 5.5 times more likely after correction with the Bayesian Information criterion.

Appendix D.2 shows the results in terms of the hit rate or the false alarm rate. The hit rate did not differ among the PI conditions, while the false alarm rate was higher in the *Repeated Condition*. Both the hit rate and the false alarm rate was greater for Set-Size 20 than for Set-Size 12, suggesting that participants had a greater tendency to endorse items for the larger set-size.

### Discussion

Experiment 2 revealed three crucial results. First, as in Experiment 1, PI is reliably observed with spatially distributed items. Second, unlike in Experiment 1 and Makovski’s (2016) experiments, PI is reliably observed with a pool-size of 21 — when the set-sizes were increased to 12 or 20 items (compared to 8 items in Experiment 1 and in Makovski’s (2016) experiments). As the total pool-size was similar, one would not expect PI to be observed if participants had encoded item-location combinations, as they should not be sensitive to the set-size. The results of Experiment 2 thus clearly rule out that PI is driven exclusively by the total pool-size, and thus by interference between item-location combinations. Participants are thus sensitive to both the set-size and the total pool-size, ruling out that they primarily encode item-location combinations rather than simple items.

Second, neither the performance in the *Unique Condition* nor the strength of PI differed between the set-sizes 12 and 20. While the absence of a set-size effect in the *Unique Condition* is consistent with earlier results (e.g., Endress & Potter, 2014a), one would expect a further increase in PI between Set-Sizes 12 and 20 if PI strength is monotonically related to the waiting time between simple items.

However, these results have three straightforward and mutually non-exclusive (post-hoc) interpretations. First, the set-size varied across trials within a block; plausibly, PI might just track the mean waiting time between recurrences of an item; after all, the relative difficulty of the *Repeated Condition* compared to the *Unique Condition* resides in the fact that it is difficult to determine whether a given test item occurred in the current as opposed to previous trials, and not in the number of items presented in a given trial. If so, an additional set-size effect should be observed when the set-size is manipulated across blocks and not across trials.

Second, the dose-effect relation between recurring items and PI might simply not be linear. For example, Endress and Szabó (2017) noted that people need to search through some memory space and that this memory space might be more or less crowded. To the extent that memory spaces function similarly to other representational spaces, they pointed out that visual crowding occurs only if items are closer than some critical distance (e.g., Pelli, Palomares, & Majaj, 2004; van den Berg, Roerdink, & Cornelissen, 2007); if items are closer than that distance, recognition is impaired, but not if items are more separated. If a similar situation holds for memory, the strength of PI (as measured by the waiting time between consecutive occurrences of the same item) would need to be greater than some critical value for interference effects to be observable, but, beyond this critical value, the strength of PI might increase much more slowly.

Third, and relatedly, if spatial information acts predominantly as a retrieval cue rather than as a part of the (short-term) memory representations, the usefulness of a retrieval cue likely depends on the overall level of interference. In the party analogy above, when trying to decide who among our friends was present at a specific party, we might use the party as a retrieval cue to run a memory search through our friends. However, the memory search will not succeed if we see our friends too often; if we see them every day, it might be hard to decide if we have seen them at some party *as well*, and the difficulty of this memory search does not necessarily show a linear relationship with the frequency of meeting friends.

Be that as it might, the combined results of Experiments 1 and 2 show that PI is readily observed for spatially distributed items and that observers are sensitive to both the total pool-size and the set-size. These results thus rule out that participants rely on item-location combinations, but are consistent with the possibility that spatial cues might act as retrieval cues as long as PI is not too strong.

## Experiment 3: The role of spatial distribution and predictability

In Experiment 3, I explored the effects of the spatial distribution of the memory items on memory performance and susceptibility to PI, respectively. I kept the set-size and the pool-size constant (with a high ratio between the set-size and the pool-size) and presented items either centrally on the screen or on an imaginary circle. Critically, when items were presented on an imaginary circle, they appeared either in a predictable sequence of locations or in a random sequence of locations.

### Materials and methods

In all trials, participants viewed a sequence of 15 objects. Critically, across blocks, items were presented either at the center of the screen (in the *Center Condition*), on an imaginary circle where items proceeded in a clockwise direction (in the *Circle-Ordered Condition* ) or on an imaginary circle where items positions on the circle were chosen at random (in the *Circle-Random Condition*). In both circle conditions, the position of the first sample item was randomly chosen.

Each block comprised a sub-block with trial-unique items and a sub-block where items were drawn from a pool of 16 items in total.

The order of blocks and sub-blocks was counterbalanced across participants. Specifically, I used all 6 possible orders of the three blocks; further, for half of the participants, the *Repeated Condition* (within each block) preceded the *Unique Condition*, while the order was reversed for the remaining participants, leading to 12 counterbalancing conditions in total.

Each of the 6 blocks (3 location conditions × 2 PI conditions) comprised 48 trials; participants were offered the opportunity to take a break every 24 trials. Experiment 3 comprised 288 trials in total.

### Results

I first analyze the results in terms of the performance in the *Unique Condition* and in terms of the Cost of PI and then in terms of the raw accuracy in the *Unique Condition* and the *Repeated Condition*, respectively.

#### Analyses of memory vs. susceptibility to PI

**Fig. 7:**
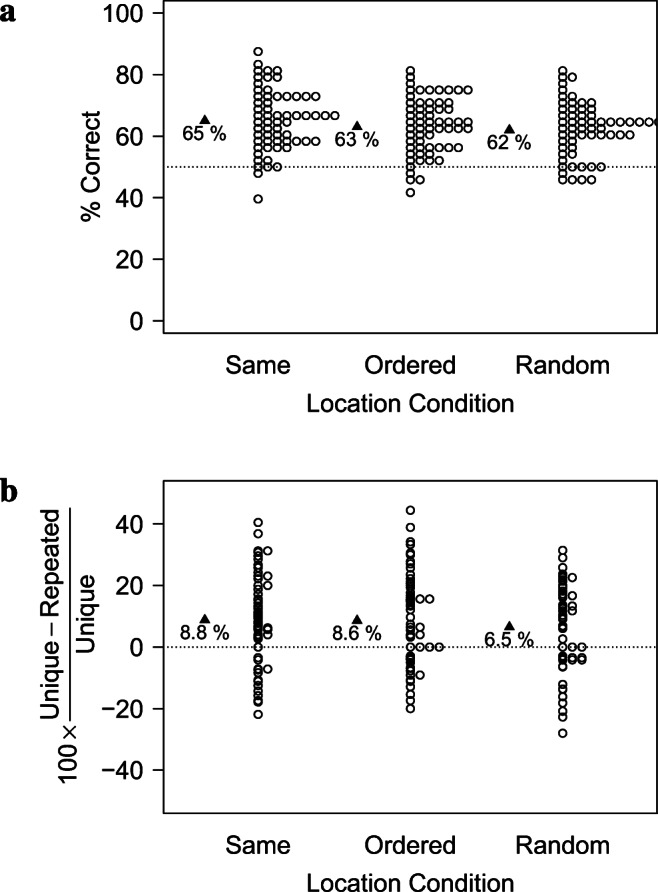
Results of Experiment 3 in terms of the raw performance in the *Unique Condition* (a) and the relative Cost of PI (b). While performance in the *Unique Condition* was better when items were presented at the center of the screen than when they were spatially distributed, the Cost of PI was relatively unaffected by these spatial manipulations

As shown in Fig. 7 and Table 7, memory performance in the *Unique Condition* was better when items were presented at the center of the screen compared to the two conditions where they were spatially distributed, with no difference between the latter two conditions. In contrast, the Cost of PI was not affected by the *Location Condition* manipulation at all.

**Table 7:** Descriptive statistics in terms of raw accuracy and Cost of PI in Experiment 3. *p*_*Wilcoxon*_ indicates the *p* values of a Wilcoxon test against the chance levels of 50% (accuracy) and 0 (Cost of PI), respectively

*PI Condition*	*Location Condition*	N	*M*	*SD*	*SE*	*p*_*Wilcoxon*_	Cohen’s *d*
Accuracy							
Unique	Center	60	0.655	0.099	0.013	<.001	1.557
Unique	Circle - Random	60	0.624	0.088	0.011	<.001	1.413
Unique	Circle - Ordered	60	0.631	0.089	0.012	<.001	1.459
Repeated	Center	60	0.586	0.080	0.010	<.001	1.072
Repeated	Circle - Random	60	0.576	0.083	0.011	<.001	0.922
Repeated	Circle - Ordered	60	0.570	0.079	0.010	<.001	0.879
Cost of PI							
–	Center	60	0.091	0.143	0.019	<.001	0.638
–	Circle - Ordered	60	0.083	0.150	0.019	<.001	0.554
–	Circle - Random	60	0.066	0.141	0.018	0.001	0.468

This impression was confirmed by pairwise Wilcoxon tests among the conditions. As shown in Table 8, performance in the *Unique Condition* was better in the *Center* Condition than in the *Circle Random* Condition and than in the *Circle Ordered* Condition. In contrast, performance did not differ significantly among the two circle conditions, nor were there any statistically significant differences in terms of the Cost of PI.

**Table 8:** Pairwise Wilcoxon test for the Location Conditions in Experiment 3

Measure	*Location Condition* 1	*Location Condition* 2	V	*p*	*CI*
Unique	Center	Circle - Ordered	1074	0.049	4.76e-05, 0.0625
Unique	Center	Circle - Random	994	0.030	3.41e-05, 0.0625
Unique	Circle - Ordered	Circle - Random	805	0.772	− 0.0209, 0.0313
Cost of PI	Center	Circle - Ordered	978	0.645	− 0.039, 0.0624
Cost of PI	Center	Circle - Random	1092	0.194	− 0.0127, 0.0798
Cost of PI	Circle - Ordered	Circle - Random	949	0.632	− 0.041, 0.0669

In sum, memory (as operationalized in the *Unique Condition*) was impaired when items were spatially distributed, with little difference between predictable and random positions. In contrast, the strength of PI was largely unaffected by these manipulations.

I further investigate these results in two ways. First, I split the *Location Condition* predictor into *two* predictors, indicating whether the spatial location of items was predictable (i.e., in the Center and the Ordered conditions) or not (in the Random condition), and whether the items were spatially distributed or not. I fit models to the data (separately for the *Unique Condition* and the Cost of PI) that included both predictors and models that included only a single one. (The models for the accuracy data treated data as binomial, while the models for the Cost of PI treated it as Gaussian, though similar results are obtained when both models assume Gaussian distributions.) Following this, I removed one of the predictors to establish whether it significantly contributed to the model likelihood.

As shown in Table 9, removing *Spatial Distribution* from the model significantly impaired the model fit for the *Unique Condition*, but not for the Cost of PI. In contrast, *Location Predictability* did not contribute to the model likelihood for either measure. These analyses thus confirm that memory is affected by spatially distributing memory items irrespective of whether their positions are predictable or not, while PI seems invariant under such manipulations.

**Table 9:** Contributions of the predictability and the spatial distribution of the items. While the spatial distribution contributed to the model likelihood in terms of the accuracy in the *Unique Condition*, predictability of item locations did not. In contrast, the *Cost of PI* was unaffected by either manipulation

Measure	Removed Predictor	*χ*^2^	Df	*p*
Accuracy (Unique Condition)	Spatial Distribution	3.763	1	0.052
Accuracy (Unique Condition)	Location Predictability	0.272	1	0.602
Cost of PI	Spatial Distribution	0.128	1	0.721
Cost of PI	Location Predictability	0.494	1	0.482

To provide direct evidence for the null hypothesis, I investigated these results using likelihood ratios (Glover & Dixon, 2004) corresponding to these contrasts. Specifically, for each participant, I calculated the difference (1) between performance in the predictable conditions and performance in the unpredictable ones, and (2) between performance in the spatially distributed conditions and performance with central presentation of the items. I then asked if a model fitting a non-zero value to these differences would fit the data better than a model where the differences were fixed to zero, assuming the data were normally distributed and correcting for the different numbers of parameters (i.e., whether an intercept was fit) using Bayesian Information Criterion and the Akaike Information Criterion.

For the *Unique Condition*, the likelihood ratios strongly favored the alternative hypothesis in the case of Distributivity (likelihood ratios in favor of the null hypothesis: 0.128 (Akaike Information Criterion) and 0.341 (Bayesian Information Criterion)). In contrast, they were ambiguous as to whether *Location Predictability* affected memory performance (likelihood ratios in favor of the null hypothesis: 0.658 (Akaike Information Criterion) and 1.746 ((Bayesian Information Criterion)).

For the Cost of PI, there was evidence favoring the null hypothesis: For both the alternative hypothesis that the Cost of PI is affected by (1) the presence of spatial information (likelihood ratios in favor of the null hypothesis: 2.032 (Akaike Information Criterion) and 5.394 (Bayesian Information Criterion)) and (2) its predictability (likelihood ratios in favor of the null hypothesis: 1.731 (Akaike Information Criterion) and 4.597 (Bayesian Information Criterion)).

In other words, memory performance was impaired when items were spatially distributed, replicating Makovski’s 2016results. However, memory performance was fairly unaffected by the predictability of the item locations (but see below).

In contrast, the Cost of PI was unaffected by either factor. The latter result is surprising because the Circle-Random Condition requires extremely rapid shifts in attention, and yet does not increase the susceptibility to PI. However, this result is consistent with previous finding that only memory but not the strength of PI is affected by temporal manipulations (Endress & Siddique, 2016) or manipulations of visual or executive attention (Endress, in preparation).

#### Analysis in terms of accuracy

**Fig. 8:**
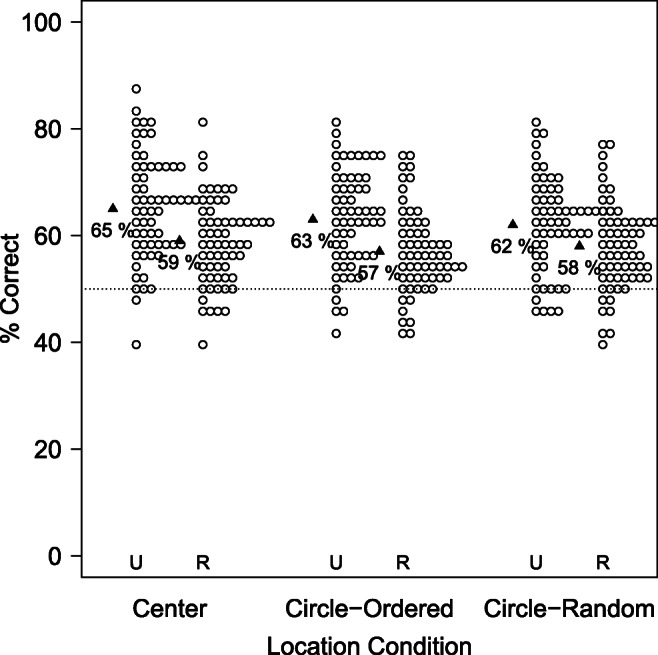
Results of Experiment 3 in terms of raw accuracy. Performance was better in the *Unique Condition* than in the *Repeated Condition* and when items were presented at the center for the screen than when they were spatially distributed. In contrast, the strength of the PI effect seemed unaffected by the spatial distribution of the items

As shown in Fig. 8, performance was better in the *Unique Condition* than in the *Repeated Condition*. Performance was also better in the *Center Condition* than in the *Circle-Random Condition*, while the *Circle-Ordered Condition* seemed to yield an intermediate performance.

I sought to confirm this impression using a generalized linear mixed model with the within-subject predictors *PI Condition* and *Location Condition*, treating the trial-by-trial accuracy as a binary random variable. Following Baayen et al., (2008), I then removed the interaction term from the model as it did not contribute to the model likelihood. I also included the order of the PI conditions, the order of the Location Conditions and their pairwise interactions (but no third or fourth order interactions) as fixed factor predictors as well as the Block Number as a random factor predictor. Only the interaction between the *Location Condition* and the *Location Condition Order* contributed to the model likelihood.

As shown in Table 10, performance was better in the *Unique Condition* than in the *Repeated Condition* and marginally better in the *Center Condition* than in the *Circle-Random* Condition. There were also a number of interactions between the *Location Condition* and the *Location Condition Order* that were examined in more detail in Appendix D.3 in terms of hit and false alarm rates.

**Table 10:** Results of a generalized linear mixed model for Experiment 2. For the *PI Condition*, the reference level was the *Unique Condition*; for the *Location Condition*, the reference level was the *Center Condition*. For the *Location Order*, the reference level was Ordered-Random-Center

Effect	Estimate	Std. Error	*CI*	*t*	*p*
*PI Condition*: Repeated	− 0.250	0.031	− 0.311, − 0.189	− 8.030	0.000
*Location Condition*: Circle-Ordered	− 0.004	0.094	− 0.188, 0.179	− 0.046	0.963
*Location Condition*: Circle-Random	− 0.181	0.093	− 0.364, 0.000773	− 1.952	0.051
*Location Condition*: Center × *Location Order*: Ordered-Center-Random	0.040	0.125	− 0.205, 0.285	0.319	0.749
*Location Condition*: Circle-Ordered × *Location Order*: Ordered-Center-Random	0.040	0.125	− 0.205, 0.286	0.322	0.748
*Location Condition*: Circle-Random × *Location Order*: Ordered-Center-Random	0.262	0.125	0.0171, 0.507	2.097	0.036
*Location Condition*: Center × *Location Order*: Random-Ordered-Center	− 0.017	0.128	− 0.268, 0.234	− 0.131	0.896
*Location Condition*: Circle-Ordered × *Location Order*: Random-Ordered-Center	0.144	0.129	− 0.109, 0.396	1.116	0.264
*Location Condition*: Circle-Random × *Location Order*: Random-Ordered-Center	0.209	0.128	− 0.0415, 0.459	1.635	0.102
*Location Condition*: Center × *Location Order*: Random-Center-Ordered	0.216	0.129	− 0.0368, 0.469	1.675	0.094
*Location Condition*: Circle-Ordered × *Location Order*: Random-Center-Ordered	− 0.071	0.128	− 0.322, 0.179	− 0.558	0.577
*Location Condition*: Circle-Random × *Location Order*: Random-Center-Ordered	0.301	0.128	0.05, 0.553	2.350	0.019
*Location Condition*: Center × *Location Order*: Center-Ordered-Random	− 0.028	0.128	− 0.279, 0.223	− 0.218	0.827
*Location Condition*: Circle-Ordered × *Location Order*: Center-Ordered-Random	− 0.137	0.128	− 0.387, 0.114	− 1.070	0.285
*Location Condition*: Circle-Random × *Location Order*: Center-Ordered-Random	− 0.041	0.127	− 0.29, 0.208	− 0.323	0.747
*Location Condition*: Center × *Location Order*: Center-Random-Ordered	0.245	0.129	− 0.0082, 0.499	1.897	0.058
*Location Condition*: Circle-Ordered × *Location Order*: Center-Random-Ordered	0.013	0.128	− 0.239, 0.264	0.098	0.922
*Location Condition*: Circle-Random × *Location Order*: Center-Random-Ordered	0.297	0.128	0.0461, 0.549	2.319	0.020

Hit rates were lower in the *Unique Condition* than in the *Repeated Condition*, and lower in the *Circle-Random Condition* than in either the *Circle-Ordered Condition* or the *Center Condition*, with no difference between the latter two conditions. However, the pattern of interactions with the *Location Condition Order* suggests that hit rates in the *Circle-Random Condition* tended to somewhat higher when it was presented as the first block, while hit rates in the *Circle-Ordered Condition* tended to be somewhat lower when the Circle-Random Condition was presented before the Circle-Ordered Condition. False alarm rates were higher in the *Repeated* compared the *Unique Condition*.

Taken together, these results provide no evidence for the hypothesis that spatially distributing items affects the strength of PI. They reveal indistinguishable PI effects across the *Location Conditions* that seem to be primarily carried by an increase in false alarm rates.

In contrast, spatially distributing items seems to affect memory. Accuracy as well as hit rates were reduced in the *Circle-Random Condition*, while the relative performance in the *Circle-Ordered Condition* seemed to depend on the order in which the Location Conditions were presented. The numeric performance reduction in the Circle-Ordered Condition might thus partially reflect (strategic) order effects.

### Discussion

The results of Experiment 3 suggest that the susceptibility to PI is not affected by spatial information. This is consistent with the interpretation of Experiments 1 and 2 that spatial information provides retrieval cues when PI is not too strong, but that it does not lead to memory for item-location combinations.

However, an alternative interpretation is that people cannot encode more than a few item-location bindings. This possibility might take four different (but not mutually exclusive) forms, but, as I will argue below, all of them are either refuted by the data or point to a limited role of spatial information in the resolution of PI.

First, observers might spontaneously bind items to location (though they still need to allocate attention to the items; Treisman and Gelade, 1980), but with too many locations, each location becomes less distinct and thus less useful. For example, when eight objects are presented on a circle, a resolution of 45^∘^ is sufficient, while a resolution of 24^∘^ would be required with 15 objects. Critically, however, if item-location bindings occur spontaneously, the items should still be bound to some *approximate* location, even if the resolution is not sufficient to encode the location precisely. As a result, the effect of PI should still be reduced relative to when items are presented at the same central location, which was clearly not the case. The results of Experiment 3 thus rule out that the lack of an effect of spatial distribution is due to a limited precision of the location encoding system.

Second, item-location bindings might occur spontaneously, but observers might only be able to maintain a limited number of bindings in memory, similarly to how some authors propose that we can maintain only a limited number of items in memory (e.g., Cowan, 2001; Fukuda et al.,, 2010; Hartshorne, 2008; Luck & Vogel, 1997; Rouder et al.,, 2008; Zhang & Luck, 2008). If so, one would predict that observers retain the *last* bindings. If binding occurs spontaneously, they keep binding items to locations as they experience more item-location combinations; but as they cannot retain all of them, earlier bindings are overwritten by later item-location combinations.

I tested the predictions of this account in two ways. (The detailed results are presented in Appendix C). In the first analysis, I consider the *Cost of PI* in the *Circle-Ordered Condition*. One would expect a main effect of the sequential position: the *Cost of PI* should be reduced in later positions compared to earlier positions. However, there was no trace of such an effect, and, in fact, likelihood ratio analysis provided evidence in favor of the null hypothesis.

In the second analysis, I consider the raw accuracy in the *Unique* and the *Repeated* Conditions of the *Center Condition* and the *Circle-Ordered Condition*, again as a function of the sequential position of the items. The critical prediction is a triple interaction between these factors. If spatial information reduces PI, one would expect an interaction between the *Location Condition* and the *PI Condition*. However, if observers cannot encode more than a few item-location bindings, this double interaction should be strongest for the most recent sequential positions, resulting in a triple interaction between the *Location Condition*, the *PI Condition* and the *Sequential Position*. Again, these predictions were unsupported.

The third way in which observers might face difficulties when encoding an excessive number of item-location bindings assumes that they automatically stop binding items to locations when there are more than a handful of locations. While the current experiments do not rule out this possibility, it does raise the question of why observers would not use a more approximate representation of the locations instead of forgoing this source of information altogether, and if an automatic item-location binding system really estimates the expected number of bindings even before the start of each trial to decide whether the bindings should be encoded.

Fourth, and relatedly, item-location binding might *not* occur spontaneously; rather, observers might strategically avoid encoding such bindings under some conditions. While this possibility is not ruled out by the data, it would suggest that location information does not necessarily reduce the effects of PI.

Taken together then, the possibility that people might face difficulties when encoding more than a few item-location bindings is either contradicted by the data or seems to suggest a limited role of spatial information for PI resolution.

## Analysis by recency

**Fig. 9:**
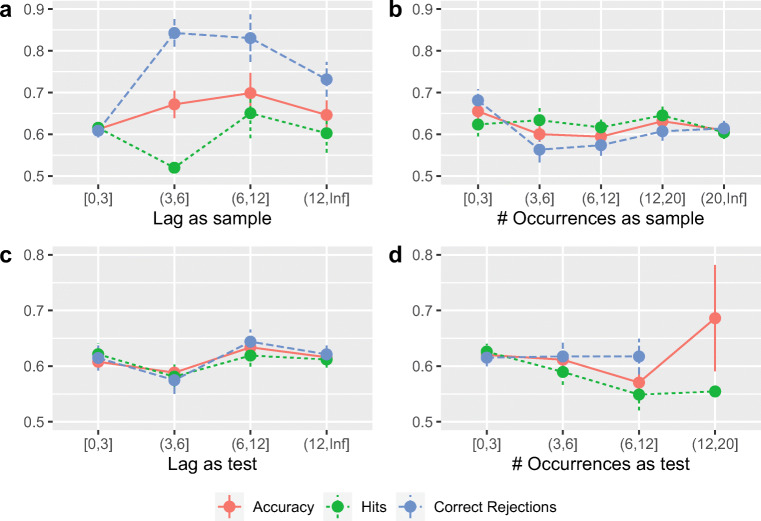
Performance in terms of accuracy, hit rates and correct rejection rates as a function of four measures that might affect the strength of PI: (a) the number of trials elapsed since the current test item appeared as a *sample item*, (b) the total number of prior trials on which the current test item has appeared as a *sample item*, (c) the number of trials elapsed since the current test item appeared as a *test item*, and (d) the total number of prior trials on which the current test item has appeared as a *test item*. The x-axis labels use interval notation; for example, (3,6] is the set of all numbers strictly greater than 3 and up to (and including) 6. Performance is more affected when test items were previously encountered as *sample* items than when they previously occurred as *test* items. Accuracy and correct rejection rates were decreased when a test item has occurred more recently or more of often as a *sample* item (while hit rates were increased). In contrast, recent or frequent *test* items seem to have a much weaker effect on performance

So far, I analyzed the effects of PI by comparing performance in high-PI blocks with higher Set-Size-to-Pool-Size ratios to low-PI blocks with lower Set-Size-to-Pool-Size ratios. However, it also possible to analyze the effects of recent occurrences of items *within* blocks.^12^ To do so, I combined all trials from all blocks in all experiments where items were presented in a predictable order on a circle, excluding all blocks with trial-unique items as well as the *Center* and *Circle-Random* conditions from Experiment 3. For each trial, I then calculated 4 measures that might affect the strength of PI: (i) the number of trials *elapsed* since the current test item appeared as a *sample item* (“*Lag as sample*” in Fig. 9a), (ii) the *total number* of prior trials on which the current test item has appeared as a *sample item* (“*# Occurrences as sample*” in Fig. 9b), (iii) the number of trials *elapsed* since the current test item appeared as a *test item* (“*Lag as test*” in Fig. 9c), and (iv) the *total number* of prior trials on which the current test item has appeared as a *test item* (“*# Occurrences as test*” in Fig. 9b).

While the current experiments were not designed with this analysis in mind, Fig. 9 shows a number of interesting trends. First, performance (in terms of accuracy, hit rates and correct rejections) seems much more affected when test items were previously encountered as *sample* items than when they previously occurred as *test* items. Accuracy and correct rejection rates were reduced when a test item has occurred more recently or more of often as a *sample* item (while hit rates were increased). In contrast, recent or frequent *test* items seem to have a much weaker effect on performance.

These impressions were confirmed in separate Generalized Linear Mixed Models, predicting performance (in terms of accuracy, hit rates and correct rejection rates) based on the four recency and frequency measures above. Before fitting the models, I set the lag of items that had never occurred as (sample or test items) to the maximum lag in the other trials, and transformed all four measures using the function log(1 + *x*) to make their distribution less left-skewed. “Success” on individual trials was treated as a binary random variable.

As shown in Table 11, accuracy was improved when the test items had appeared less recently and less frequently as *samples*. In contrast, the recency of the test item as *test item* did not affect accuracy, while the frequency as *test items* had only a smaller effect.

**Table 11:** Results of a generalized linear mixed model the effect of item recency and frequency on accuracy, hit rates and correct rejection rates. See Fig. 9 for a description of the different measures

Effect	Estimate	Std. Error	*CI*	*t*	*p*
Accuracy					
Lag as sample	0.212	0.042	0.129, 0.295	5.013	0.000
# Occurrences as sample	− 0.064	0.018	− 0.099, − 0.0299	− 3.660	0.000
Lag as test	0.014	0.014	− 0.0129, 0.0401	1.005	0.315
# Occurrences as test	− 0.055	0.024	− 0.101, − 0.00799	− 2.296	0.022
Hits					
Lag as sample	− 0.137	0.060	− 0.254, − 0.0196	− 2.289	0.022
# Occurrences as sample	− 0.087	0.026	− 0.138, − 0.0358	− 3.327	0.001
Lag as test	0.030	0.020	− 0.00934, 0.0685	1.489	0.136
# Occurrences as test	− 0.184	0.035	− 0.253, − 0.116	− 5.262	0.000
Correct Rejections					
Lag as sample	0.666	0.074	0.522, 0.81	9.043	0.000
# Occurrences as sample	− 0.071	0.027	− 0.124, − 0.0173	− 2.597	0.009
Lag as test	0.010	0.021	− 0.0309, 0.0506	0.474	0.636
# Occurrences as test	0.020	0.037	− 0.0516, 0.0921	0.553	0.580

Hit rates were somewhat increased for test items that were recent or frequent sample items, while the frequency as test items had a less pronounced effect. In contrast, hit rates were *reduced* for items that had frequently occurred as *test items*, maybe because participants change their response criterion for items that occur frequently as test items.

The estimates for hit rates were relatively small compared to those found for correct rejection rates. Correct rejections were much lower for test items that had appeared recently or frequently as *sample items*, while occurrences as *test items* did not appear to have an effect.

While the current experiments were not designed to address these issues, these results allow for three tentative conclusions. First, at least in the current paradigm, PI might act predominantly by increasing false alarm rates when test items have been encountered recently or frequently as *samples*. Second, prior occurrences as *test items* seem to have a less pronounced effect. One possible explanation is that test items are presented for much longer than sample items; they also presented in a distinct context because they are not surrounded by other items. This might allow participants to better encode the context of the test items, which, in turn, makes them less likely to interfere on subsequent trials.

Third, the frequency of test items as test items had a detrimental effect on hit rates. A possible explanation is that participants might adjust their response criterion for items that occur frequently as test items. While this would explain why, in Experiment 1, hit rates are higher in the *Unique Condition* compared to the Pool-Size-9 Condition, it would not explain this opposite effect in Experiments 2 and 3. As a result, more targeted experiments are needed to confirm these conclusions.

## General discussion

Why are some WM experiments more sensitive to PI than others? One possibility is that spatial information protects memory from PI. Indeed, previous research has found only limited evidence for PI when items were spatially distributed and presented sequentially (though PI effects were reliable in very similar experiments where items were presented simultaneously; Makovski, 2016). This leads to three hypotheses about why PI is reduced when items are spatially distributed. First, observers might store item-location combinations; as there are many more item-location combinations than simple items, this should reduce PI among the relevant memory representations. Second, the availability of spatial cues might change the task demands, in that they introduce retrieval cues for memory searches. Third, some WM paradigms might be intrinsically less sensitive to PI than others.

The current experiments revealed two critical results. First, PI is readily observed with spatially distributed items, at least when it is sufficiently strong. In Experiment 1, performance was impaired when sequences of 8 items were drawn from a total pool of 9 items compared to a condition with trial-unique items. In contrast, with a total Pool-Size of 21 items, performance was undistinguishable from performance with trial-unique items, but the sensitivity to PI reemerged in Experiment 2 when the set-size was increased to 12 or 20 (in different trials). Further, a combined analyses of all three experiments revealed reduced performance when the test items had occurred as *sample* items on recent trials, in contrast to similar analysis of color-change change detection experiments (e.g., Hartshorne, 2008; Lin & Luck, 2012; Makovski & Jiang, 2008). In contrast, prior or recent occurrences as *test* items had a much smaller effect, possibly because it is easier to encode the context of the test items. Accordingly, PI was most visible in an increase in false alarm rates. However, participants also had reduced hit rates for test items that frequently occurred as test items, suggesting that, under some conditions, participants also adjust their response criteria.

Second, observers are sensitive to the ratio between the set-size and the pool-size. This constrains the role of spatial information for short-lived forms of memory, because observers should be sensitive to the set-size only if they store simple items, but not if the primary memory representations are item-location combinations; in the latter case, they should only be sensitive to the total pool-size, but not the set-size.

However, when the set-size further increased from 12 to 20, no further changes were observed, neither for memory performance nor for the Cost of PI. In the case of memory performance in the *Unique Condition*, this result is consistent with Endress and Potter’s (2014a) finding that performance in the *Unique Condition* is relatively unaffected by the set-size, at least for larger set-sizes.^13^ In the case of the Cost of PI, as mentioned in the discussion of Experiment 2, the lack of a further increase in the Cost of PI has three mutually non-exclusive explanations. First, PI might just track the mean frequency of recurrence of memory items (which does not change across set-sizes if set-sizes are chosen randomly within a block). Second, the relationship between the waiting time between two occurrences of an item and the observable strength of PI might be non-linear. Third, and relatedly, retrieval cues might become less useful with stronger background PI.

Finally, Experiment 3 investigated the role of spatial information of PI more directly. While, in line with previous experiments (Makovski, 2016), memory performance suffered when items were spatially distributed, spatially distributing item had no effect at all on the strength of PI. Interestingly, depending on the analysis, spatially distributing items impaired memory irrespective of whether the item locations were predictable or not, though the reduced performance for spatially distributed items in predictable locations might partially reflect ordering effects.

Taken together, the present results show that, just like other forms of memory, visual WM is susceptible to PI. This, in turn, raises the question of why visual WM as implemented in change detection experiments is relatively *insensitive* to PI, especially given how superficially similar the current experiments are to change detection experiments: in both types of paradigms, participants view arrays that exceed their WM capacity (e.g., up to 12 items in Luck & Vogel, 1997; experiments), and, at least in tasks where participants have to respond to single items (e.g., Balaban et al.,, 2019), they can successfully complete the task by just binding these items to the current temporal context (i.e., the current trial). The current results rule out another potential explanation of these discrepant results, namely that the role of spatial information is to make items more distinct by allowing observers to encode item-location combinations. However, spatial information might still have other effects.

One such possibility is that spatial cues provide participants with retrieval cues that allow them to perform memory searches, similarly to how, in the party analogy above, we can search our memory to discriminate friends who were at a party from friends who were not (provided that the friends are sufficiently familiar to begin a memory search).

A second possibility is that spatial information might allow observers to encode entire displays as configurations of items by binding together the items in the displays. If so, visual WM limitations might reflect encoding rather than memory limitations (Tsubomi et al.,, 2013; see also Fukuda & Vogel, 2009; Fukuda & Vogel, 2011). The memory representations are still likely to be susceptible to PI, but the PI effects might be relatively minor if the limitations due to encoding are much more pronounced than any limitations due to PI. This hypothesis would be in line with previous suggestions that an important function of WM is to create temporary bindings (e.g., Bateman & Birney, 2019; Oberauer et al.,, 2008), though the bindings in the case of change detection experiments would be much more perceptual than those in earlier non-visual WM experiments.

A third and mutually non-exclusive possibility is that change detection experiments are relatively insensitive to PI because they reflect attentional encoding processes rather than memory per se, which is arguably a classic theory about WM capacity (e.g., Cowan, 2005). In fact, “WM” limitations can be observed even when objects are in full view: Tsubomi et al., (2013) found similar change detection performance irrespective of whether the sample array was followed by a blank retention interval or whether the sample array remained visible until the test item.

Further, WM capacity estimates derived from change detection experiments correlate with the maximum number of items people can apprehend without counting (i.e., their subitizing range; Piazza et al.,, 2011), which, in turn, are thought to be related to attentional processes (e.g., Trick & Pylyshyn, 1994). If so, one would expect the underlying processes to be relatively *insensitive* to PI as observers can allocate attention on each trial anew (even though long-term memory can affect attention as well; Fan and Turk-Browne, 2016; Kerzel & Andres, 2020).

Be that as it might, the present experiments confirm that WM experiments with real-world objects are sensitive to PI even when the items are spatially distributed, in contrast to change detection experiments with simple features (e.g., Balaban et al.,, 2019; Hartshorne, 2008; Lin & Luck, 2012; Makovski & Jiang, 2008). This contrast raises the question of whether all WM experiments index the same cognitive processes.

## Figures and Tables

**Fig. 10:**
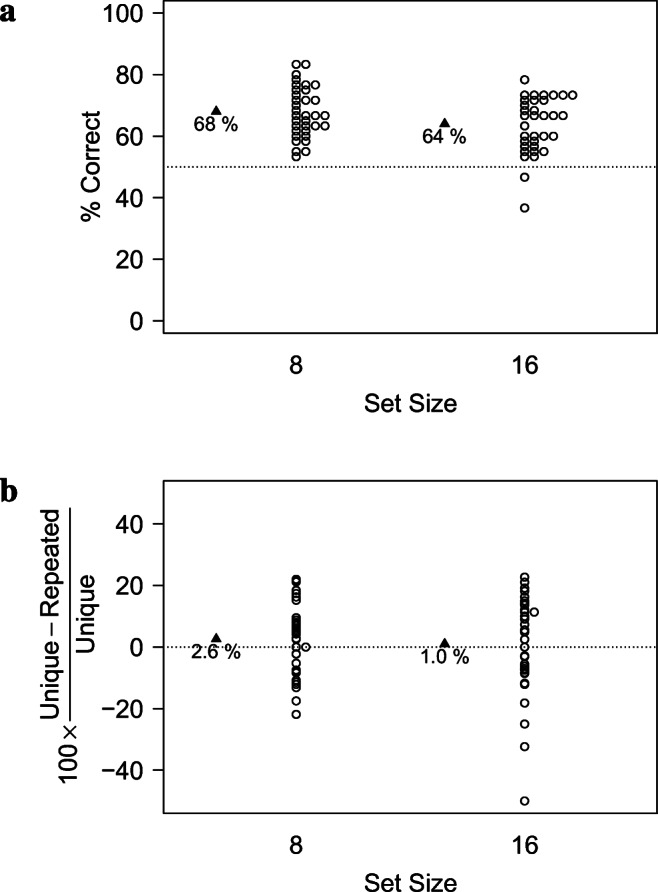
Results of the Pilot Experiment in terms of the accuracy in the *Unique Condition* (a) and the relative *Cost of PI* (b)

**Fig. 11:**
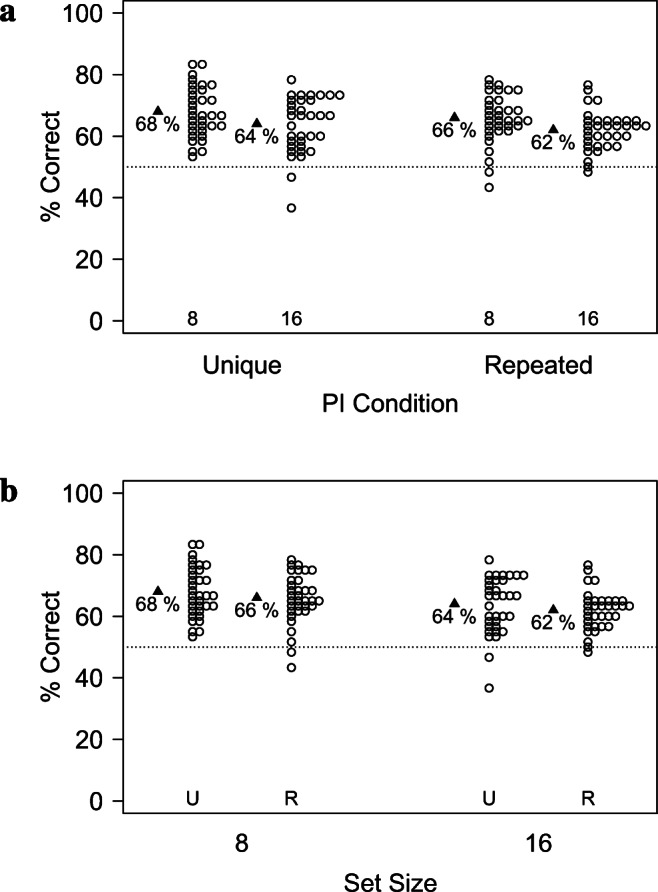
Results of the Pilot Experiment in terms of raw accuracy. U and R represent the Unique and the Repeated Conditions, respectively, Both figures show the same data, grouped by the *PI Conditions* (a) or the *Set-Sizes* (b)

**Fig. 12:**
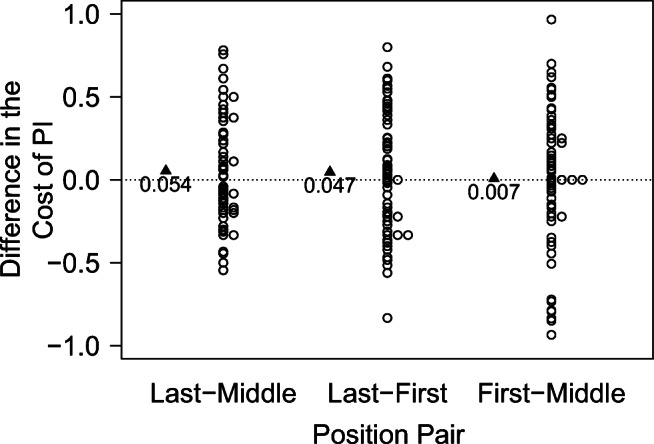
Differences between Sequential Positons in the *Circle-Ordered* Condition of Experiment 3

**Fig. 13:**
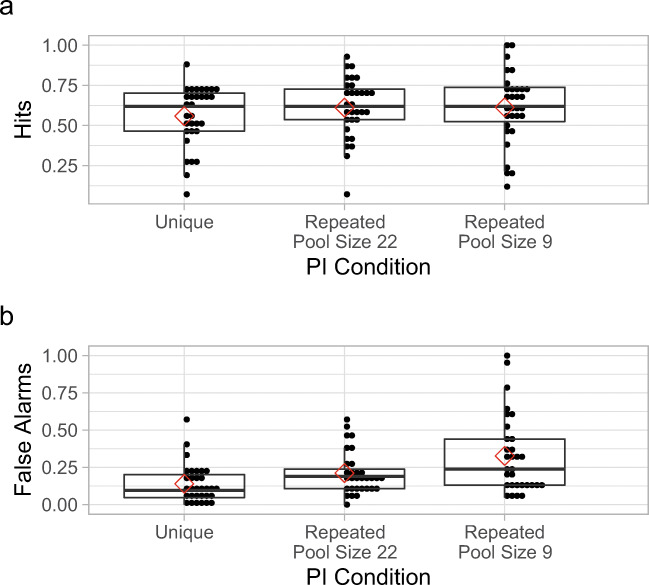
Results of Experiment 1 in terms of hits and false alarms. Each dot represents a participant, the diamond represents the sample mean, while the thick line represents the median

**Fig. 14:**
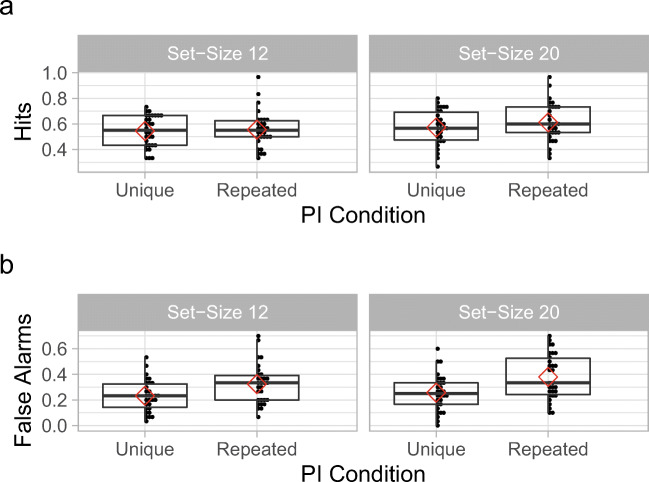
Results of Experiment 2 in terms of hits and false alarms. Each dot represents a participant, the diamond represents the sample mean, while the thick line represents the median

**Fig. 15:**
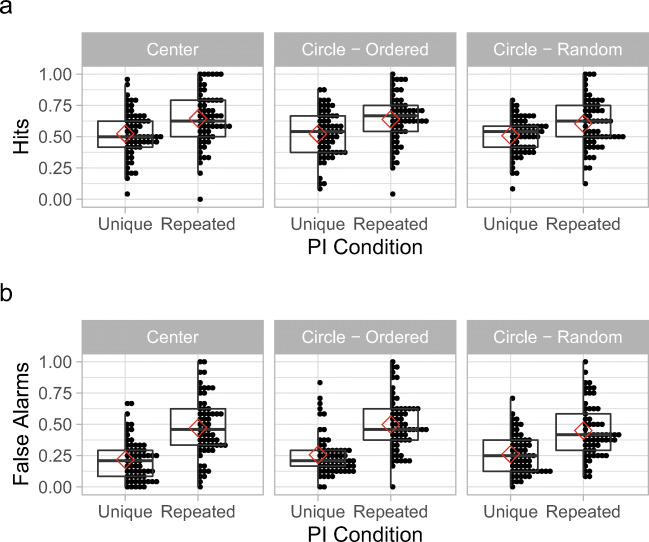
Results of Experiment 3 in terms of hits and false alarms. Each dot represents a participant, the diamond represents the sample mean, while the thick line represents the median

## Appendix A: Formulae for waiting times

Let the set-size *S* be the number of pictures presented on each trial and let *T* be the total number of pictures presented in the entire (block of an) experiment. The probability that a given picture appears as a sample (in a sample sequence or in a sample array) is thus *p* = *S*/*T* and the probability of a lag of *N* trials between successive occurrences of a sample picture is thus *P*(lag = *N*) = *p*(1 − *p*)^*N*^,

Based on these definitions, one can easily derive some further properties of the waiting time.

### **Claim 1** (Waiting time)

The probability of waiting *at least* *N* trials for the next occurrence of a picture is *P*(lag ≥ *N*) = (1 − *p*)^*N*^.

### *Proof*

By noting that *P*(lag ≥ *N*) = 1 − *P*(lag < *N*), we can apply the geometric series formula and obtain

$$ P(\text{lag} \geq N) = 1- \sum\limits_{k=0}^{N-1} p (1 - p)^{k} = 1 - p \frac{1 - (1-p)^{N}}{1 - (1-p)} = (1-p)^{N}. $$

□

### **Claim 2** (Mean waiting time)

The mean lag is between two occurrences of a picture $\frac {1-p}{p}$ trials.

### *Proof*

I first note that we can drop the first term in the series: $\langle \text {lag}\rangle = {\sum }_{N = 0}^{\infty } N P(\text {lag} = N) = {\sum }_{N = 1}^{\infty } N P(\text {lag} = N) = p {\sum }_{N = 1}^{\infty } N (1-p)^{N}$, which is a polylogarithm of order − 1. We thus obtain

$$ \langle\text{lag}\rangle = p \frac{1 - p}{(1-(1-p))^{2}} = \frac{1-p}{p} = \frac{1}{p } - 1 $$

□

### **Claim 3** (Waiting time for test items)

The probability of occurrence as a test item does not depend on the set-size.

### *Proof*

The probability of an item occurring as a test item is the sum of the probability of it occurring as an *old* test item and as a *new* test item (i.e., a foil):

$$ \begin{array}{@{}rcl@{}} P(\text{test item}) &=& P(\text{test item} | \text{old trial}) P(\text{old trial})\\ && + P(\text{test item} | \text{new trial})P(\text{new trial}). \end{array} $$

An item can be an *old* test item if (i) it is selected as a sample item, (ii) it is not in the first or the last position, and (iii) if it is selected as a test item:

$$ P(\text{test item} | \text{old trial})= \frac{S}{T} \frac{S-2}{S} \frac{1}{S-2} = \frac{1}{T}. $$

Likewise, an item can be a new test item if (i) it is not selected as a sample item, and (ii) it is selected as a test item:

$$ P(\text{test item} | \text{new trial}) = \left( 1 - \frac{S}{T}\right) \frac{1}{T-S} = \frac{1}{T}. $$

In sum, the probability of an item occurring as a test item is thus given by $P(\text {test item} | \text {old trial}) P(\text {old trial}) + P(\text {test item} | \text {new trial})P(\text {new trial}) = 2 \frac {1}{T} \frac {1}{2} = \frac {1}{T}$ and does not depend on the set-size. □

## Appendix B: Pilot Experiment: The role of the set-size (2)

The pilot experiment was similar to Experiment 2 (testing the role of the set-size), except that the set-sizes were 8 or 16 and that items were drawn from a total pool of 17 items.

### B.1 Results and discussion

I first analyze the results in terms of the performance in the *Unique Condition* and in terms of the Cost of PI and then in terms of the raw accuracy in the *Unique Condition* and the *Repeated Condition*, respectively.

**B.1.1. Analyses of memory vs. susceptibility to PI**

As shown in Fig. 10 and Tables 12 and 13, performance in the *Unique Condition* was well above chance for both Set-Sizes, while the Cost of PI was not significantly different from zero for either Set-Size, probably because, in the *Repeated Condition*, the set-sizes were too small relative to the size of the pool from which items were drawn. In fact, the set-sizes in Experiment 2 were 12 and 20 for a pool-size of 21, while the set-sizes in the Pilot Experiment were 8 and 16 for a pool-size of 17. The ratio between the average set-size and the pool-size was thus somewhat higher in Experiment 2 (0.76; 0.57 for the smaller set-size) compared to the Pilot Experiment (0.7; 0.47 for the smaller set-size). This corresponds to average lags between two occurrences of an item of .3125 and .417 trials, respectively.

**Table 12:** Descriptive statistics in terms of raw accuracy for the Pilot Experiment. *p*_*W**i**l**c**o**x**o**n*_ indicates the *p* values of a Wilcoxon test against the chance level of 50%

*PI Condition*	*Set-Size*	N	*M*	*SD*	*SE*	*p*_*W**i**l**c**o**x**o**n*_	Cohen’s *d*
Repeated	8	32	0.655	0.083	0.015	<.001	1.88
Repeated	16	32	0.620	0.065	0.011	<.001	1.85
Unique	8	32	0.678	0.080	0.014	<.001	2.21
Unique	16	32	0.635	0.093	0.017	<.001	1.44

**Table 13:** Descriptive statistics in terms of the Cost of PI for the Pilot Experiment. *p*_*W**i**l**c**o**x**o**n*_ indicates the *p* values of a Wilcoxon test against the chance level of 0

*Set-Size*	N	*M*	*SD*	*SE*	*p*_*W**i**l**c**o**x**o**n*_	Cohen’s *d*
8	32	0.029	0.116	0.020	0.217	0.247
16	32	0.005	0.164	0.029	0.439	0.029

Further, a paired Wilcoxon test revealed that performance in the *Unique Condition* was significantly better for the smaller set-size, *V* = 308.5, *p* = 0.05, *C**I*_.95_ = -5.3e-05, 0.0834, maybe mirroring Endress and Potter’s (2014a) finding that performance in the absence of PI drops from small set-sizes to larger set-sizes, with a limited change in performance for larger set-sizes. In contrast, there was no difference between the set-sizes in terms of the Cost of PI, *V* = 259, *p*= 0.837, *C**I*_.95_ = − 0.0624, 0.09.

#### B.1.2. Analysis in terms of accuracy

As shown in Fig. 11 and Table 12, performance in Terms of raw accuracy differed somewhat across the *Set-Size*, but the effect of *PI Condition* seemed small. This was confirmed by a generalized linear mixed model with the within-subject predictors *PI Condition* and *Set-Size*, treating the trial-by-trial accuracy as a binary random variable. Following Baayen et al., (2008), I then removed the interaction term from the model as it did not contribute to the model likelihood. The results are shown in Table 14

**Table 14:** Results of a generalized linear mixed model for the Pilot Experiment. I used the *Unique Condition* and the *Set-Size* of 8 as the reference levels of the two predictors

Effect	Estimate	Std. Error	*CI*	*t*	*p*
*PI Condition*: Repeated	− 0.083	0.048	− 0.177, 0.011	− 1.73	0.084
*Set-Size*: 16	− 0.173	0.048	− 0.267, − 0.0789	− 3.60	0.000

While performance was significantly worse for the *Set-Size* 16, the difference between the *Unique* and the *Repeated* condition was only marginal.

## Appendix C: The Cost of PI across serial positions

A possible limitation of Experiment 3 is that observers might face difficulties when encoding more than a few item-location bindings because later bindings overwrite earlier bindings. I analyze the predictions of this account in two ways, one involving the *Cost of PI* and one the raw accuracy.

In the first analysis, I consider the *Cost of PI* in the *Circle-Ordered Condition*. One would expect a main effect of the sequential position: the *Cost of PI* should be reduced in later positions. (Due to violations of normality, I will actually calculate pairwise differences rather than an ANOVA). As described in the Methods section above, the serial positions are two sequence-initial positions, two sequence-medial positions and two sequence-final positions. For “new” responses, sequential positions were randomly assigned to the items.

In the second analysis, I consider the raw accuracy (on a trial-by-trial basis) in the *Unique* and the *Repeated* Conditions of the *Center Condition* and the *Circle-Ordered Condition*, again as a function of the sequential position of the items. The critical prediction is a triple interaction between these factors. If spatial information reduces PI, one would expect an interaction between the *Location Condition* and the *PI Condition*. However, if observers cannot encode more than a few item-location bindings, this double interaction should be strongest for the most recent sequential positions, resulting in a triple interaction between the *Location Condition*, the *PI Condition* and the *Sequential Position*.

### C.1 Analyses in terms of the susceptibility to PI

In the analyses below, I excluded one participant whose *Cost of PI* for the initial positions differed by more than 4.5 standard deviations from the mean. Further, as shown in Table 15, some cells showed deviation from normality. The analyses below are thus based on pairwise differences assessed by Wilcoxon tests.

**Table 15:** Cells in the Serial Position analysis for Experiment 3 where a violation of normality was detected by a Shapiro-Wilk test when performance was measured in terms of the Cost of PI

Sequential Position	Location Condition	W	*p*	*p* ≤ .05
First	Center	0.903	0.000	***
Middle	Circle - Ordered	0.941	0.006	**
First	Circle - Ordered	0.947	0.012	*

As shown in Table 16, the present data do not support the prediction that the *Cost of PI* is most pronounced towards the beginning of the sequences. In fact, numerically at least, it is more pronounced for later positions. However, as shown in Table 17 and Fig. 12, these numeric trends are not statistically reliable, as no pairwise difference between sequential positions reaches significance.

**Table 16:** Descriptive statistics in terms of *Cost of PI* of the different sequential positions (top 3 rows), and in terms of differences of the *Cost of PI* across sequential positions (bottom 3 rows)

	*N*	*M*	*SD*	*SE*
Cost of PI				
First	59	0.054	0.305	0.040
Middle	59	0.046	0.283	0.037
Last	59	0.098	0.210	0.027
Differences				
First-Middle	59	0.007	0.425	0.055
Last-First	59	0.044	0.375	0.049
Last-Middle	59	0.051	0.322	0.042

**Table 17:** Pairwise Wilcoxon test for the differences in the *Cost of PI* across Sequential Positions for the *Circle-Ordered Condition* from Experiment 3. The last two columns show the likelihood ratios for the *null* hypothesis for these differences after correction with the Akaike Information Criterion (AIC) and the Bayesian Information Criterion (BIC)

				*AIC*	*BIC*
*Difference*	*V*	*p*	*CI*	in favor of null	
Last–Middle	984	0.459	− 0.0531, 0.135	1.37	3.61
Last–First	943	0.357	− 0.0657, 0.148	1.95	5.12
First–Middle	859	0.622	− 0.0972, 0.145	2.90	7.61

To provide evidence for the null hypothesis, I computed, for each participant, the pair-wise differences between the *Cost of PI* in the different sequential positions (e.g., the difference between the *Cost of PI* for sequence-initial positions and sequence-medial positions). For each difference, I then compared two linear models with Gaussian noise with a mean of zero and a standard deviation estimated from the data. The null model set the intercept term to zero (i.e., there was no data fitting on top of estimating the variance), while the alternative model fitted the intercept term on top of estimating the variance. I then compared these models using likelihood ratios, adjust for the different number of parameters (i.e., the intercept term) using the Akaike Information Criterion and the Bayesian Information Criterion (Glover and Dixon, 2004).

As shown in Table 17, this analysis favored the null hypothesis. For example, the null hypothesis was about 5 times more likely than the alternative hypothesis the *Cost of PI* differed between the initial and the final position when correcting with the Bayesian Information Criterion, and about twice as likely when correcting with the Akaike Information Criterion. As a result, we can exclude that people spontaneously encode item-position bindings, and that earlier bindings get overwritten by later bindings.

### C.2 Analysis in terms of accuracy

In the second serial position analysis, I used a generalized linear mixed model with the within-subject predictors *PI Condition*, *Location Condition* and *Sequential Position* (two initial, medial, and final position, respectively). Sequential positions for “new” test items were assigned pro-forma, but, of course, new items has no real sequential position. In this analysis, I treated the data as binary. Following Baayen et al., (2008), I then removed all interaction terms from the model as they did not contribute to the model likelihood. The critical three-way interaction did not contribute to the model likelihood. In fact, the Akaike Information Criterion favored the reduced model by a factor of 55, while the Bayesian Information Criterion favored the reduced model by a factor of 7 × 10^7^, though these values are not necessarily interpretable as likelihood ratios in a generalized linear mixed model.

As shown in Table 18, performance was better in the *Unique Condition* than in the *Repeated Condition*, confirming the effect of PI; it was better in the *Center Condition* and in the *Circle-Random Conditions*, confirming that spatially distributing items has a cost for memory; and it was better for the final positions than for either initial or medial positions. In line with previous results, I thus observed a recency effect on overall accuracy, but recency did not affect the strength of PI.

**Table 18:** Results of a generalized linear mixed model for Experiment 3. For the *PI Condition*, the reference level was the *Unique Condition*, for the *Location Condition*, the reference level was the *Center Condition*, and for the *Sequential Position*, the reference level were the final positions. The table shows only those effects that contributed to the model likelihood

Effect	Estimate	Std. Error	CI	*t*	*p*
*PI Condition*: Repeated	− 0.277	0.039	− 0.352, − 0.201	− 7.17	0.000
*Location Condition*: same	0.087	0.039	0.0114, 0.163	2.26	0.024
*Sequential Position*: middle	− 0.269	0.048	− 0.362, − 0.176	− 5.66	0.000
*Sequential Position*: first	− 0.266	0.048	− 0.359, − 0.172	− 5.59	0.000

For completeness, Table 19 shows the results of the full model. Critically, the triple interaction is not significant.

**Table 19:** Results of a generalized linear mixed model for Experiment 3. For the *PI Condition*, the reference level was the *Unique Condition*, for the *Location Condition*, the reference level was the *Center Condition*, and for the *Sequential Position*, the reference level were the final positions. The table shows the results of the full model

Effect	Estimate	Std. Error	CI	*t*	*p*
*PI Condition*: Repeated	− 0.372	0.097	− 0.562, − 0.183	− 3.846	0.000
*Location Condition*: same	− 0.029	0.099	− 0.224, 0.165	− 0.297	0.766
*Sequential Position*: middle	− 0.381	0.097	− 0.571, − 0.192	− 3.940	0.000
*Sequential Position*: first	− 0.460	0.096	− 0.649, − 0.271	− 4.772	0.000
*PI Condition*: Repeated ×					
*Location Condition*: same	0.047	0.137	− 0.221, 0.315	0.345	0.730
*PI Condition*: Repeated ×					
*Sequential Position*: middle	0.156	0.134	− 0.108, 0.419	1.159	0.247
*PI Condition*: Repeated ×					
*Sequential Position*: first	0.179	0.134	− 0.0835, 0.442	1.337	0.181
*Location Condition*: same ×					
*Sequential Position*: middle	0.141	0.137	− 0.127, 0.41	1.031	0.303
*Location Condition*: same ×					
*Sequential Position*: first	0.257	0.137	− 0.0117, 0.525	1.874	0.061
*PI Condition*: Repeated × *Location Condition*: same ×					
*Sequential Position*: middle	− 0.150	0.190	− 0.523, 0.222	− 0.791	0.429
*PI Condition*: Repeated × *Location Condition*: same ×					
*Sequential Position*: first	− 0.099	0.190	− 0.472, 0.274	− 0.519	0.604

## Appendix D: Analyses in terms of hits and false alarms

Hit and false alarm rates were analyzed using similar generalized linear mixed models as the accuracy data above, treating responses in each trial as binary random variables. Unless otherwise stated, the fixed and random factors were the same as for the accuracy data, except that the data was restricted to “old” and “new” trials, respectively. I also included the block order and its interactions with the other predictors as a fixed factor predictors, but, except in Experiment 3, they did not contribute to the model likelihood.

### D.1 Experiment 1: The role of PI strength

As shown in Table 20 and Fig. 13, the hit rate was slightly increased for Pool-Sizes 22 and 9 compared to the *Unique Condition*, with no difference between the latter two conditions. In contrast, the false alarm rate tracked the strength of PI; false alarm rates were greater Pool-Size 9 than for Pool-Size 22; false alarm rates in the *Unique Condition* were lower than for either of these Pool-Sizes.

**Table 20:** Results of a generalized linear mixed model for Experiment 1, separately for hits and false alarms. I used the *Unique Condition* as the reference level for the *Pool-Size*

Effect	Estimate	Std. Error	*CI*	*t*	*p*
Hits — all trials					
*Pool-Size* 22	− 0.047	0.062	− 0.168, 0.0732	− 0.770	0.441
*Pool-Size* 9	− 0.313	0.060	− 0.431, − 0.195	− 5.191	0.000
Hits—Unique Condition excluded					
*Pool-Size* 9	0.007	0.086	− 0.161, 0.176	0.087	0.931
False Alarms—all trials					
*Pool-Size* 22	0.561	0.110	0.345, 0.777	5.090	0.000
*Pool-Size* 9	1.241	0.106	1.03, 1.45	11.725	0.000
False Alarms—Unique Condition excluded					
*Pool-Size* 9	0.700	0.098	0.508, 0.892	7.152	0.000

### D.2 Experiment 2: The role of the set-size

As shown in Table 21 and Fig. 14, the hit rate was higher for Set-Size 20 than for Set-Size 12, but did not differ between the Repeated and the Unique Conditions. In contrast, the false alarm rate was much higher in the *Repeated Condition* than in the *Unique Condition*, but also increased for Set-size 20 compared to Set-Size 12.

**Table 21:** Results of a generalized linear mixed model for Experiment 2, separately for hits and false alarms. I used the *Unique Condition* as the reference level for the *PI Condition* and Set-Size 12 as the reference level for the *Set-Size*

Effect	Estimate	Std. Error	*CI*	*t*	*p*
Hits					
*PI Condition*: Repeated	0.108	0.068	− 0.0265, 0.242	1.57	0.116
*Set-Size*: 20	0.178	0.068	0.0436, 0.312	2.60	0.009
False Alarms					
*PI Condition*: Repeated	0.547	0.076	0.398, 0.696	7.21	0.000
*Set-Size*: 20	0.208	0.075	0.06, 0.356	2.75	0.006

These results suggest that the effect of PI was mainly carried by an increase in false alarm rates, but that participants also had a greater tendency to endorse items for larger set-sizes.

### D.3 Experiment 3: The role of spatial information

As shown in Table 22 and Fig. 15, hit rates were lower in the *Unique Condition* than in the *Repeated Condition*, and lower in the Circle-Random Condition than in either the Circle-Ordered Condition or the Center Condition, with no difference between the latter two conditions. However, the pattern of interactions with the Block Order suggested that hit rates in the Circle-Random Condition tended to somewhat higher when it was presented as the first block, while hit rates in the Circle-Ordered Condition tended to be somewhat lower when the Circle-Random Condition was presented before the Circle-Ordered Condition.

**Table 22:** Results of a generalized linear mixed model for Experiment 3, separately for hits and false alarms. For the PI Condition, the reference level was the *Unique Condition*. For the Location Condition, the reference level was the Center Condition when it was present (top) and the Circle-Ordered Condition when the Center Condition was removed (bottom)

Effect	Estimate	Std. Error	*CI*	*t*	*p*
Hits — all trials					
*PI Condition*: Repeated	0.492	0.045	0.403, 0.58	10.881	0.000
*Location Condition*: Circle-Ordered	0.164	0.135	− 0.101, 0.429	1.214	0.225
*Location Condition*: Circle-Random	− 0.329	0.134	− 0.592, − 0.0675	− 2.465	0.014
*Location Order*: Ordered-Center-Random	0.346	0.293	− 0.23, 0.921	1.178	0.239
*Location Order*: Random-Ordered-Center	− 0.175	0.299	− 0.761, 0.411	− 0.584	0.559
*Location Order*: Random-Center-Ordered	0.087	0.302	− 0.505, 0.679	0.288	0.773
*Location Order*: Center-Ordered-Random	0.021	0.299	− 0.565, 0.608	0.072	0.943
*Location Order*: Center-Random-Ordered	0.302	0.300	− 0.286, 0.889	1.007	0.314
*Location Condition*: Circle-Ordered × *Location Order*: Ordered-Center-Random	− 0.190	0.189	− 0.561, 0.181	− 1.006	0.315
*Location Condition*: Circle-Random × *Location Order*: Ordered-Center-Random	0.114	0.187	− 0.253, 0.481	0.608	0.543
*Location Condition*: Circle-Ordered × *Location Order*: Random-Ordered-Center	0.141	0.190	− 0.232, 0.514	0.742	0.458
*Location Condition*: Circle-Random × *Location Order*: Random-Ordered-Center	0.553	0.189	0.182, 0.924	2.923	0.003
*Location Condition*: Circle-Ordered × *Location Order*: Random-Center-Ordered	− 0.382	0.198	− 0.769, 0.00492	− 1.935	0.053
*Location Condition*: Circle-Random × *Location Order*: Random-Center-Ordered	0.340	0.197	− 0.0468, 0.727	1.723	0.085
*Location Condition*: Circle-Ordered × *Location Order*: Center-Ordered-Random	− 0.209	0.191	− 0.583, 0.164	− 1.098	0.272
*Location Condition*: Circle-Random × *Location Order*: Center-Ordered-Random	0.149	0.189	− 0.222, 0.521	0.789	0.430
*Location Condition*: Circle-Ordered × *Location Order*: Center-Random-Ordered	− 0.487	0.190	− 0.86, − 0.114	− 2.558	0.011
*Location Condition*: Circle-Random × *Location Order*: Center-Random-Ordered	0.086	0.190	− 0.286, 0.457	0.451	0.652
Hits — Circle-Center Condition excluded					
*PI Condition*: Repeated	0.464	0.055	0.356, 0.572	8.441	0.000
*Location Condition*: Circle-Random	− 0.491	0.134	− 0.754, − 0.228	− 3.656	0.000
*Location Order*: Ordered-Center-Random	0.147	0.275	− 0.392, 0.687	0.536	0.592
*Location Order*: Random-Ordered-Center	− 0.042	0.281	− 0.592, 0.508	− 0.150	0.881
*Location Order*: Random-Center-Ordered	− 0.270	0.283	− 0.824, 0.284	− 0.955	0.340
*Location Order*: Center-Ordered-Random	− 0.186	0.281	− 0.737, 0.364	− 0.663	0.507
*Location Order*: Center-Random-Ordered	− 0.181	0.280	− 0.73, 0.369	− 0.644	0.520
*Location Condition*: Circle-Random × *Location Order*: Ordered-Center-Random	0.304	0.187	− 0.0626, 0.671	1.626	0.104
*Location Condition*: Circle-Random × *Location Order*: Random-Ordered-Center	0.410	0.190	0.0386, 0.782	2.163	0.031
*Location Condition*: Circle-Random × *Location Order*: Random-Center-Ordered	0.714	0.196	0.33, 1.1	3.648	0.000
*Location Condition*: Circle-Random × *Location Order*: Center-Ordered-Random	0.356	0.190	− 0.0156, 0.728	1.877	0.060
*Location Condition*: Circle-Random × *Location Order*: Center-Random-Ordered	0.571	0.189	0.2, 0.941	3.018	0.003
False Alarms – all trials					
*PI condition*: Repeated	1.132	0.049	1.04, 1.23	22.985	0.000
*Location Condition*: Circle-Ordered	0.196	0.144	− 0.0857, 0.477	1.363	0.173
*Location Condition*: Circle-Random	0.052	0.145	− 0.231, 0.335	0.360	0.719
*Location Order*: Ordered-Center-Random	0.278	0.340	− 0.389, 0.945	0.817	0.414
*Location Order*: Random-Ordered-Center	− 0.167	0.350	− 0.852, 0.519	− 0.477	0.633
*Location Order*: Random-Center-Ordered	− 0.534	0.355	− 1.23, 0.161	− 1.505	0.132
*Location Order*: Center-Ordered-Random	0.123	0.348	− 0.559, 0.805	0.353	0.724
*Location Order*: Center-Random-Ordered	− 0.184	0.350	− 0.869, 0.502	− 0.525	0.600
*Location Condition*: Circle-Ordered × *Location Order*: Ordered-Center-Random	− 0.214	0.196	− 0.599, 0.171	− 1.087	0.277
*Location Condition*: Circle-Random × *Location Order*: Ordered-Center-Random	− 0.375	0.198	− 0.764, 0.0143	− 1.888	0.059
*Location Condition*: Circle-Ordered × *Location Order*: Random-Ordered-Center	− 0.207	0.206	− 0.61, 0.197	− 1.003	0.316
*Location Condition*: Circle-Random × *Location Order*: Random-Ordered-Center	0.108	0.206	− 0.294, 0.511	0.527	0.598
*Location Condition*: Circle-Ordered × *Location Order*: Random-Center-Ordered	0.364	0.215	− 0.0582, 0.786	1.690	0.091
*Location Condition*: Circle-Random × *Location Order*: Random-Center-Ordered	0.237	0.217	− 0.188, 0.663	1.093	0.274
*Location Condition*: Circle-Ordered × *Location Order*: Center-Ordered-Random	0.009	0.200	− 0.383, 0.402	0.047	0.962
*Location Condition*: Circle-Random × *Location Order*: Center-Ordered-Random	0.192	0.201	− 0.202, 0.586	0.953	0.341
*Location Condition*: Circle-Ordered × *Location Order*: Center-Random-Ordered	− 0.025	0.205	− 0.427, 0.376	− 0.124	0.901
*Location Condition*: Circle-Random × *Location Order*: Center-Random-Ordered	− 0.041	0.206	− 0.446, 0.363	− 0.200	0.842

False alarm rates were higher in the Repeated compared the *Unique Condition*. Taken together, these results suggest that PI is primarily carried by an increase in false alarm rates, and that, in line with the accuracy analyses, only memory but not PI is sensitive to spatial manipulations. However, while hit rates were clearly reduced in the Circle-Random Condition, it is unclear to what extent they are reduced in the Circle-Ordered Condition compared to the Center Condition, in that the relatively low performance in the Circle-Ordered Condition in the accuracy analyses might partially reflect different response strategies due to different block orderings.

## Footnotes

1 The susceptibility to interference might also be determined by the number of bindings in which an item is embedded (Humphreys, Bain, & Pike, 1989; Humphreys, Wiles, & Dennis, 1994).

2 Prior experience might not only lead to proactive interference, but also to proactive facilitation. For example, Oberauer et al., (2017) first trained participants on associating silhouettes of objects with colors (drawn from a continuous color space). Following this, they completed a WM task using these silhouettes. In each trial, they viewed three silhouettes. After a retention interval, they were cued with one of these silhouettes shown in white and had to report its color on a color wheel. Compared to novel items, WM performance was better when the color of the silhouette matched its earlier color, but there was no difference between novel items and silhouettes whose color mismatched the earlier colors. In other words, there was proactive facilitation, but no proactive interference. However, these results might be due to the specific WM paradigm used. Participants needed to recall continuously varying colors and thus stimuli that cannot be encoded categorically and thus are poorly remembered in the short-term (e.g., Olsson & Poom, 2005) and in the long-term (e.g., Brown and Lenneberg, 1954; Hasantash & Afraz, 2020). Accordingly, LTM recollection for the initial shape-color associations was rather poor, and certainly much weaker than memory for meaningful items after much less exposure (e.g., Brady, Konkle, Alvarez, & Oliva, 2008; Standing, Conezio, & Haber, 1970). It is thus possible that stronger interference effects might have been found if stimuli had been used that are more reliably encoded in LTM. In contrast, given that the strength of even weak memory representations increases with further exposure (e.g., Endress & Potter, 2014b; Melcher, 2001, 2006; Pertzov, Avidan, & Zohary, 2009; Thunell & Thorpe, 2019), these LTM traces might be sufficient to generate proactive facilitation, while being too weak to trigger a response on their own as would be required for proactive interference. A second possible explanation of these results relies on the interplay between memory strength in WM (rather than in LTM) and a possible “gating” mechanism. This mechanism would allow participants to rely on LTM when WM representations are not sufficiently strong, but protect WM against LTM otherwise (Oberauer et al., 2017). If the initial shape-color association mismatches the association in the current trial (and WM is weak), LTM-based responses are indistinguishable from random guesses in terms of performance, and no proactive interference should ensue. In contrast, if the initial shape-color association *matches* the association in the current trial (and WM is weak), LTM-based responses enhance performance, leading to proactive facilitation.

3 In terms of memory capacity estimates, Endress and Potter (2014a) observed “capacities” of up to 30 items for sequences of 100 items, derived from Cowan’s (2001) two-high-threshold formula. However, given that the accuracy in the memory test was relatively constant across set-sizes, the capacity estimates depended on the set-size. For example, an accuracy of 75% yields a capacity estimate of 5 for set-size 10 and a capacity estimate of 10 for set-size 20. However, one would expect participants to perform at ceiling as long as the set-size remains below the capacity estimate (e.g., 30 for a set-size of 100 items), which was clearly not the case. As a result, capacity estimates are not necessarily meaningful in these experiments. This does not seem to be a specific problem of Endress and Potter’s (2014a) experiments, as performance decreases even within the putative memory capacity range in visual WM experiments as well (e.g., Schneegans & Bays, 2016).

4 Even more dramatically, in a large scale Web-based study, Hartshorne (2008) observed a memory capacity of only 2.75 on the first trial of experiment, where PI could not yet have occurred. However, given that his displays consisted of only 4 items, memory capacity estimates (e.g., Cowan, 2001; Rouder et al.,, 2008) are mathematically limited to 4, and a capacity of 2.75 corresponds to an accuracy of 84%. It is thus possible that Hartshorne (2008) would have observed larger capacity estimates if he had used larger set-sizes.

5 For example, in Treisman and Zhang (2006) and Udale et al., (2017) experiments, participants had to report feature changes irrespective of which other feature they were bound to. If the sample array contained a red square and a blue triangle, a test array containing a red triangle and a blue square would require a “no change” response because all features were already present in the sample array. (In contrast, an orange square would elicit a “change” response, as no sample object was orange.) When new feature combinations (e.g., the red triangle) are shown in the test array, participants perform better if they appear in a *new* location, presumably because feature combinations are bound into objects (e.g., Luck & Vogel, 1997), which are bound to spatial locations in turn, and the memory for the entire objects is less likely to be accessed when the objects are shown at a new location.

6 While I use terms such as time and delay to describe these effects, they do not necessarily reflect absolute times measured in seconds, but might rather be relative times measured with respect to some experimental variables such as the presentation duration.

7 More specifically, performance in the *Unique Condition* was reduced between Experiments 1 and 4, while performance in the *Repeated Condition* was identical.

8 According to the formula in the previous section, the mean waiting time is giving by $\frac {1}{p} - 1= \frac {1}{\frac {S}{ S \times T}} - 1 = T -1$

9 While this result might appear counterintuitive at first, it can be made plausible through the following observation. If the set-size, and thus the number of positions, equals the total number of items *T*, once an item (e.g., a dog) has appeared in some position, we need to wait of the order of *T* trials for it to appear again *in that position*. In contrast, if we have twice as many items in total as we have positions, and if the dog happens to be selected on every trial, we need to wait of the order of *T*/2 trials for it to reappear in a position again (since we have *T*/2 positions). However, since the dog will be selected only on half of the trials, the waiting time for it to reappear in an earlier position is again *T*. Hence, if participants represent item-location combinations, the waiting time between two occurrences of an item-location combination, and thus the strength of their PI, only depends on the total number of items, but not on the set-size.

10 This follows again from the expression for the mean waiting time derived in the previous section: $\frac {1}{p} - 1 = \frac {1}{\frac {S}{ T}} - 1 = \frac {T}{S} -1$.

11 Items will not overlap if the circles inscribing them are more distant than their diameter. The distance between two points on a circle with radius *r* is given by *d* = 2*r* sin (*α*/2) (where *α* is the angle between the two points on the circle); the diameter of the circle inscribing the objects is 2*x* (where *x* is the edge length of the square). To make sure that the pictures do not overlap, the condition *d* > 2*x* thus needs to be satisfied, or, equivalently with *N* pictures, *x* < 2 2 sin (180/*N*)*r*.

12 I am grateful to an anonymous reviewer for this suggestion.

13 In the pilot experiment presented in Appendix B, we did find an effect of Set-Size on performance in the *Unique Condition*, but this might be because the smaller set-size was relatively small.
